# Multivalent Tranexamic Acid (TXA) and Benzamidine Derivatives for Serine Protease Inhibition

**DOI:** 10.1021/acsptsci.5c00030

**Published:** 2025-05-15

**Authors:** Tanmaye Nallan Chakravarthula, Rodrigo Santillan-Rodriguez, Ziqian Zeng, Abigail Hall, Andres Prieto Trujillo, Anushri Umesh, Nathan J. Alves

**Affiliations:** a Department of Emergency Medicine, Indiana UniversitySchool of Medicine, 12250 Indiana University, Indianapolis, Indiana 46202, United States; b Weldon School of Biomedical Engineering, Purdue University, West Lafayette, Indiana 47907, United States

**Keywords:** benzamidine, enzyme inhibition, multivalency, serine proteases, tranexamic acid

## Abstract

Blood coagulation and fibrinolysis pathways involve many serine proteases in a careful equilibrium. Disruption of this hemostatic balance can cause life-threatening thromboembolic and bleeding disorders that require therapeutic intervention. Heterobivalent molecules synthesized with both benzamidine (active site serine protease inhibitor) and tranexamic acid (TXA, kringle/lysine-site inhibitor) of increasing dPEG linker lengths (dPEG_4_–dPEG_36_) were synthesized and analyzed for plasmin, thrombin, and tissue plasminogen activator (tPA) inhibition using soluble enzymatic substrates. Linker lengths greater than the active and lysine binding site separation achieved improved inhibition with plasmin and tPA due to multivalent subsite binding effects. Despite TXA being a weak active site inhibitor, homomultivalent TXA (PAMAM^8^-TXA) demonstrated strong competitive plasmin inhibition (*K*_i_ = 2.5 ± 1.8 μM) due to the statistical rebinding effect. IC_50_ values were also determined by assaying on physiologically relevant, fluorescently tagged, annular fibrin clots to capture the effect of kringle binding inhibition on fibrinolytic potential in the presence and absence of inhibitors.

## Graphical abstract

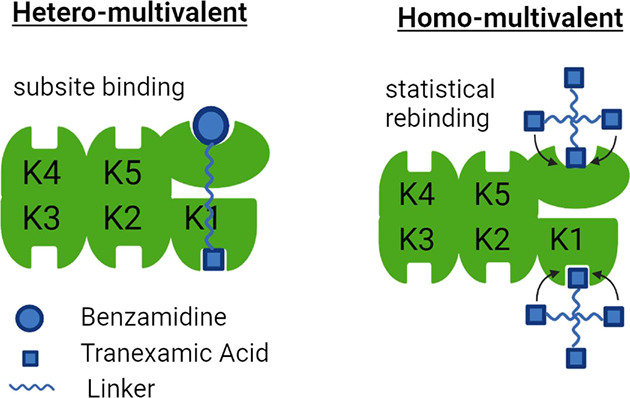

Serine proteases are a large family of proteases comprising over one-third of all proteolytic enzymes. They play a vital role in various physiological processes such as blood coagulation, fibrinolysis, digestion, and immunity.^1^ The coagulation cascade is regulated by various trypsin-like serine proteases that include factors VIIa, IXa, Xa, and XIa and thrombin (IIa).^2^ Thrombin, the key serine protease in blood coagulation, converts fibrinogen to fibrin, a major component of the blood clot, to drive clot formation.^3^ On the other hand, serine proteases such as plasmin­(ogen) and tissue plasminogen activator (tPA) are important in the fibrinolytic pathway. Plasmin degrades fibrin to drive clot digestion and is activated from its precursor (plasminogen) by plasminogen activators (PAs), such as tPA.^4^ A healthy circulatory system is maintained by a delicate balance of the coagulation and fibrinolysis pathways, and an imbalance in the activity of the serine proteases involved can cause serious thromboembolic (pathological clotting) or bleeding disorders that can require therapeutic intervention.^5^

As thrombin, plasmin, and tPA are the final enzymes involved in clot formation and digestion, they have been primary targets for treating clotting and bleeding disorders. FDA-approved treatment strategies for these disorders involve regulating serine proteases either by administering their inhibitors or by directly administering serine proteases themselves that have an opposing effect. For example, thromboembolic disorders that include deep vein thrombosis, pulmonary embolism, and myocardial infarction are treated by administering thrombin inhibitors (e.g., dabigatran) to limit further clot formation or are treated with tPA directly to promote clot digestion.^6^ Similarly, inhibiting plasmin­(ogen) or administering coagulation factors are strategies to treat bleeding disorders.^7,8^ Therefore, hemostatic balance can be achieved either by activating one pathway or by an entirely different intervention that inhibits the opposing pathway.

Trypsin-like serine proteases are characterized by an acidic aspartate residue at the bottom of the S1 binding pocket. Benzamidine, an arginine mimetic, interacts with the aspartate and is widely employed as a substructure in the design of serine protease inhibitors.^9,10^ Direct thrombin inhibitors (DTIs) such as dabigatran and argatroban are FDA-approved anticoagulants that are benzamidine/arginine derivatives that inhibit the active site of thrombin.^11,12^ Other thrombin inhibitors used clinically include bivalent direct thrombin inhibitors such as hirudins (lepirudin, desirudin, and bivalirudin) and indirect inhibitors such as heparin and low-molecular-weight heparin (LMWH). These inhibitors interact with thrombin’s exosites (exosite I or II) that are positively charged (basic) regions that regulate thrombin’s activity and specificity.^13,14^ Exosite I is involved in interaction with fibrin­(ogen) and activation of coagulation factors, whereas exosite II is responsible for platelet interactions and binding to the fibrinogen γ’ chain.^15^ Since exosites play a key role in thrombin’s interaction with substrates, cofactors, and inhibitors, anticoagulants that target the exosites are of interest to treat thrombosis.^16^ Multiple bivalent and trivalent thrombin inhibitors that target not only the active site of thrombin but also the exosites I and/or II of thrombin have been developed.^15,17^

Similar to exosites on thrombin, plasmin and tPA have kringle domains that facilitate their interaction with substrates and inhibitors. Plasmin­(ogen) has five kringle domains (K1–K5, as shown in Figure 1A) that have lysine binding sites (LBSs) that bind to lysine residues on fibrin.^18^ tPA also has kringle 2 and finger domains that mediate its interaction with fibrin.^19^ Tranexamic acid (TXA) and ε-aminocaproic acid (EACA) are FDA-approved LBS inhibitors used clinically for treating hyperfibrinolysis-associated bleeding. They are lysine analogues that mimic lysine residues on fibrin and inhibit fibrinolysis by blocking fibrin–plasmin­(ogen) interactions.^20,21^ Although TXA is a 10-fold stronger inhibitor of plasmin­(ogen) than EACA, high doses of TXA are required (1–1.5 g administered three to four times a day) to treat hyperfibrinolysis-associated bleeding events.^22^ Large clinical trials have shown that treatment with TXA significantly reduces bleeding and mortality when administered within a 3 h window. However, it can be ineffective, or even detrimental, if the treatment is delayed beyond 3 h after traumatic injury.^23,24^ Therefore, there is a need for alternative potent antifibrinolytic agents. To address this need, inhibitors that can block plasmin’s active site will be beneficial as they can reduce bleeding more rapidly. Plasmin also plays a role in a variety of additional processes that include extracellular matrix (ECM) degradation, activation of matrix metalloproteinases (MMPs), and expression of proinflammatory cytokines and also takes part in cell invasion and metastasis. Therefore, plasmin inhibitors may have a wide variety of potential applications including managing cancer and inflammatory disorders.^20,25^

**Figure 1:**
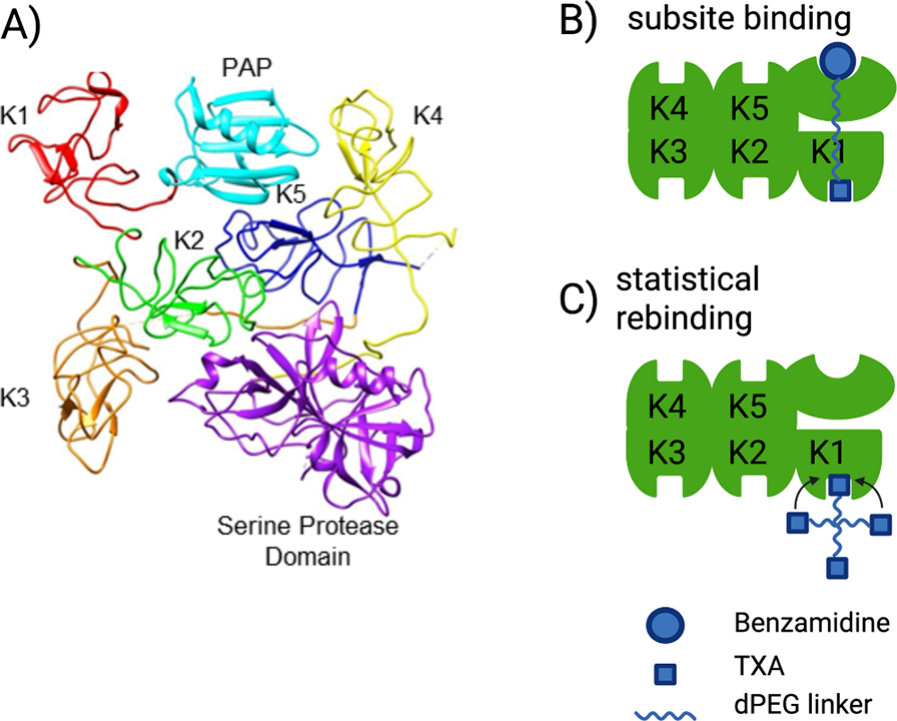
(A) Crystal structure of plasminogen (4DUR) depicting five kringle domains K1–K5, pan apple domain (PAP), and serine protease domain that has the active site. (B) Subsite binding of heterobivalent inhibitors: benzamidine to plasmin’s active site and tranexamic acid (TXA) to the lysine binding sites (LBS) on kringle domain K1. (C) Statistical rebinding of TXA to the kringle domain K1 of plasmin.

Plasmin’s *in vivo* inhibitor, α2-antiplasmin, is a bivalent plasmin inhibitor that inhibits both plasmin’s active site as well as LBS on its kringle domains. In part due to its bivalent inhibition, α2-antiplasmin exhibits a high affinity toward plasmin, resulting in its rapid and irreversible inhibition. Although there are various studies on multivalent thrombin inhibitors, very limited research has been carried out on bivalent plasmin and tPA inhibitors. In one study, Tsuda et al. tested TXA-Tyr-EACA-linker-Lys inhibitors of different linker lengths and showed that a bivalent plasmin inhibitor with a longer linker length was able to achieve bivalent inhibition similar to antiplasmin.^26^ The study presented here explores multivalent inhibition further by leveraging heterobivalent inhibitors that were engineered to simultaneously target both plasmin’s active site and LBS on kringle domains using dPEG linkers of varying lengths. For this, we utilized benzamidine as the small molecule active site inhibitor and TXA (or EACA) as LBS inhibitors to achieve the subsite binding effect of multivalency (Figure 1B).^27^ These inhibitors were also tested for their tPA and thrombin inhibition. Having observed a stronger inhibition of plasmin with multivalent benzamidine derivatives via statistical rebinding in a previous study, we also sought to evaluate the effect of valency on plasmin inhibition by homomultivalent TXA derivatives in this study (Figure 1C).^28^ In addition to these hetero- and homomultivalent inhibitors, monovalent benzamidine derivatives, TXA, and EACA were also tested and compared for their plasmin, tPA, and thrombin inhibition.

## Results and Discussion

### Serine Protease Active Site Inhibition with Benzamidine Derivatives

Benzamidine derivatives are competitive, reversible, active site inhibitors of a variety of serine proteases that include plasmin, tPA, and thrombin.^29,30^ Pentamidine, a clinically approved bivalent benzamidine for antimicrobial activity, is a competitive inhibitor of serine proteases involved in blood coagulation/fibrinolysis such as plasmin and factor Xa.^31^ Pentamidine and commercially available benzamidine derivatives benzamidine, 4-amino benzamidine, 4-carboxy benzamidine, 4-aminomethyl benzamidine (AMB), and synthesized Tri-AMB were tested for their tPA and thrombin inhibition and were compared to plasmin inhibition that was previously determined.^28,32^ Inhibition assays were performed to determine inhibition constants (*K*_i_ values) using Dixon plot analysis, and these obtained *K*_i_ values were compared across all three serine proteases (Table 1). Inhibition assays with plasmin were performed using 100–500 μM chromogenic substrate (S-2251) and 42.5 nM human plasmin over a range of inhibitor concentrations (0–300,000 μM). Plasmin activity was tracked by monitoring *p*-nitroaniline released by the hydrolysis of S-2251 at 405 nm. Inhibition assays for tPA were carried out using Chromogenix S-2288. A range of S-2288 concentrations (100–500 μM) and inhibitor concentrations (0–150,000 μM) and a fixed concentration of human tPA (75 nM) were utilized to perform inhibition assays. Similarly, the tPA activity was tracked at 405 nm by monitoring the absorbance of *p*-nitroaniline released by the hydrolysis of S-2288. Inhibition assays for thrombin utilized fluorogenic thrombin substrate III and were carried out at TSIII concentrations of 20–50 μM, inhibitor concentrations of 0–250,000 μM, and a fixed human thrombin concentration of 0.25 U/mL. Thrombin activity was determined by monitoring the fluorescent AMC tag (λ_ex_: 370 nm and λ_em_: 450 nm) released by the hydrolysis of TSIII (see Figures S1–S11). Pentamidine, a bivalent benzamidine, was the strongest inhibitor across all three serine proteases—plasmin, tPA, and thrombin—with *K*_i_ values of 2.1 ± 0.8, 43 ± 9.7, and 4.5 ± 2.3 μM, respectively, whereas AMB was the weakest benzamidine derivative with *K*_i_ values of 1074 ± 19, 5209 ± 161, and 344 ± 33 μM, respectively. In general, benzamidine derivatives have more comparable *K*_i_ values with plasmin and thrombin (<3-fold difference) and were found to be weaker inhibitors of tPA (Table 1).

**Table 1:** Inhibition Constants (*K*_i_) of Benzamidine Derivatives with Plasmin, tPA, and Thrombin

**inhibitor**	**plasmin *K***_**i**_ **(μM)** ^28,32^	**tPA *K*_i_ (μM)**	**thrombin *K*_i_ (μM)**
benzamidine	32 ± 3.0	438 ± 2.4	63 ± 12
4-amino benzamidine	51 ± 2.4	148 ± 0.9	43 ± 13
4-carboxy benzamidine	292 ± 6.5	NA^a^	151 ± 4.3
4-aminomethyl benzamidine	1074 ± 19	5209 ± 161	344 ± 33
pentamidine	2.1 ± 0.8	43 ± 9.7	4.5 ± 2.3
Tri-AMB	3.9 ± 1.7	164 ± 17	11 ± 2.0

a NA: could not test due to solubility limitations.

### Design and Synthesis of Heterobivalent Inhibitors

Heterobivalent inhibitors comprising both benzamidine and TXA that can target plasmin’s active site and lysine binding sites (LBSs) on the kringle domains simultaneously were designed. TXA has a strong affinity for the LBS on the K1 domain of plasmin with a *K*_i_ of 1.1 μM and binds weakly to the other plasmin kringle domain LBS with *K*_i_ values of ∼750 μM.^21^ Inhibitors with increasing dPEG linker lengths were explored to attain subsite binding by ensuring that sufficiently long linkers were utilized to allow for simultaneous binding to both the active site and LBS on plasmin’s K1. To achieve this goal, linkers ranging from dPEG_4_ to dPEG_36_ corresponding to ∼4–17 nm were used to determine their effect on plasmin inhibition and compare it with tPA and thrombin inhibition. To synthesize these inhibitors, AMB was first reacted with Fmoc-dPEG*x*-NHS/TFP esters (*x* = 4, 8, 12, and 36) in a mixture of DMF and PBS as shown in Scheme 1. Fmoc was deprotected using 30% piperidine in DMF, and the NH_2_-dPEG*x*-AMB obtained was purified via HPLC and confirmed with mass spectrometry. NH_2_-dPEG*x*-AMB was then reacted with Fmoc-TXA in DMF at room temperature using HBTU and DIEA. Fmoc was deprotected using 30% piperidine, the TXA-dPEG*x*-AMB product was purified on HPLC, and the final masses were again verified with mass spectrometry. EACA is also an LBS inhibitor and is ∼10-fold less potent than TXA.^33^ EACA-dPEG_4_-AMB was synthesized following the same protocol replacing Fmoc-TXA with Fmoc-EACA to determine its inhibitory effect (see Figures S12–S16).

**Scheme 1:**
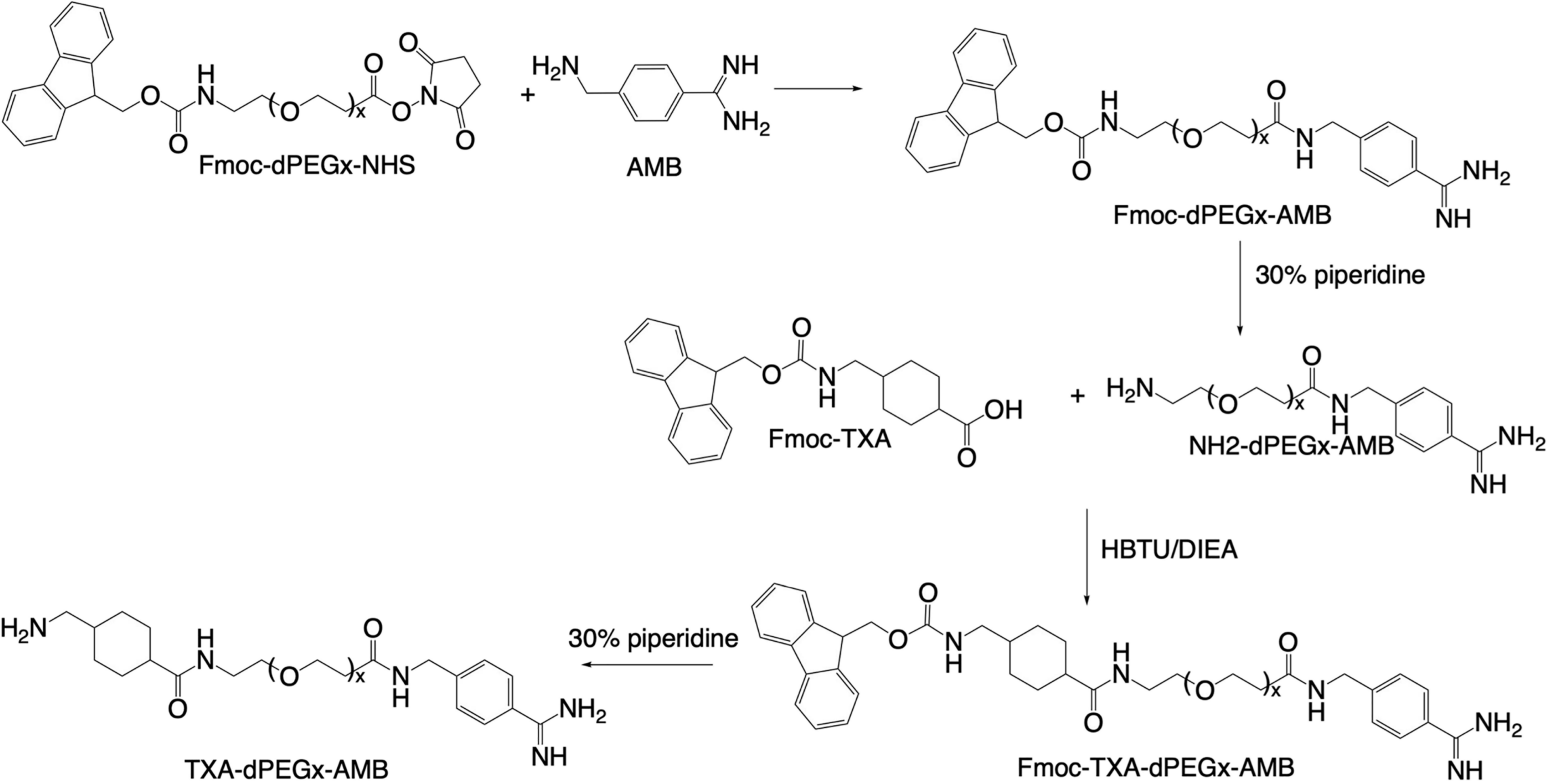
Synthesis Scheme for Heterobivalent Inhibitors TXA-dPEG*x*-AMB (*x* = 4, 8, 12, and 36)

### Inhibition of Serine Proteases with Heterobivalent inhibitors

Following the synthesis of the heterobivalent inhibitors, inhibition assays were performed to determine and compare their *K*_i_ values across plasmin, thrombin, and tPA. The *K*_i_ values are shown in Table 2 along with the calculated planar separation lengths of the inhibitors measured end-to-end using ChemDraw (version 19.0.1.). The synthesized heterobivalent inhibitors have *K*_i_ values in the range of benzamidine derivatives and are at least 5-fold more potent inhibitors of plasmin than monovalent AMB and over 100-fold more potent than monovalent TXA (see Table S1 and Figures S17–S23). TXA-dPEG_4_-AMB was the strongest heterobivalent plasmin inhibitor with a *K*_i_ of 42 ± 12 μM. The planar separation distance between LBS on K1 of plasmin and the active site was measured using Chimera and found to be ∼8 nm (see Figure S24). Hence, dPEG_4_ to dPEG_12_ linker lengths cannot achieve subsite binding at K1 as they are <8 nm in length. Therefore, inhibition decreased with increasing linker length, as seen previously with homomultivalent benzamidine inhibitors. This is likely due to a decrease in effective local inhibitor concentration and an increase in entropic penalty associated with the longer linker length.^28^ TXA-dPEG_36_-AMB, instead of being a weaker inhibitor due to longer linker length, was found to be a stronger inhibitor than TXA-dPEG_12_-AMB with a *K*_i_ value of 75 ± 4.3 μM. As anticipated, this improved inhibition was indicative of simultaneous binding to both the active site and LBS on K1. Although dPEG_36_ can achieve enhanced inhibition due to subsite binding in plasmin, higher entropic penalty due to longer linker length counteracts some of the improvement in inhibition and therefore resulted in an overall weaker inhibition than that of TXA-dPEG_4_-AMB with plasmin. The stronger inhibition observed with short heterobivalent linkers, such as TXA-dPEG_4_-AMB, results potentially from nonspecific interactions between the inhibitors and the protein surface functioning as a nonspecific avidity effect. This enhances inhibition despite the linkers not being long enough for domain-specific subsite binding. EACA-dPEG_4_-AMB was found to be a weaker inhibitor of plasmin than TXA-dPEG_4_-AMB with a *K*_i_ of 233 ± 5.0 μM due to EACA being a weaker inhibitor of plasmin than TXA. Therefore, longer linker lengths with EACA were not synthesized and tested.

**Table 2:** Inhibition Constants (*K*_i_) of Heterobivalent Inhibitors with Plasmin, tPA, and Thrombin

**inhibitor**	**length (nm)**	**plasmin *K*_i_ (μM)**	**tPA *K*_i_ (μM)**	**thrombin *K*_i_ (μM)**
TXA	0	21,370 ± 3300	122,850 ± 33,950	210,382 ± 41,400
TXA-dPEG_4_-AMB	4.0	42 ± 12	231 ± 30	14 ± 6.7
TXA-dPEG_8_-AMB	5.5	99 ± 0.9	292 ± 0.4	32 ± 3.2
TXA-dPEG_12_-AMB	7.1	207 ± 4.1	641 ± 57	46 ± 1.5
TXA-dPEG_36_-AMB	16.6	75 ± 4.3	136 ± 0.7	78 ± 1.5
EACA	0	71,150 ± 25,540	327,748 ± 50,027	65,166 ± 2907
EACA-dPEG_4_-AMB	4.0	233 ± 5.0	501 ± 32	4.0 ± 4.0

The heterobivalent inhibitors were also tested for their inhibition of tPA and thrombin using the methods described above. TXA-dPEG_36_-AMB was the strongest heterobivalent inhibitor of tPA with a *K*_i_ of 136 ± 0.7 μM instead of being the weakest due to longer linker length. This indicates a potential subsite binding effect with simultaneous binding to both the active site and LBS of tPA. Interestingly, despite not having any kringle domains, EACA-dPEG_4_-AMB was the strongest heterobivalent inhibitor of thrombin with a *K*_i_ of 4.0 ± 4.0 μM and was found to be 3-fold stronger than TXA-dPEG_4_-AMB, the strongest TXA derivative with a *K*_i_ of 14 ± 6.7 μM. Monovalent EACA was also found to be a 3-fold stronger inhibitor of thrombin than monovalent TXA. Due to the absence of kringle domains in thrombin and therefore the absence of the specific kringle-associated subsite binding effect, inhibition steadily decreased with an increase in linker length due to higher entropic penalty (see Figures S25–S38).

### Design and Synthesis of Homomultivalent TXA Inhibitors

We have previously demonstrated stronger inhibition of plasmin with higher valency multivalent benzamidine derivatives.^28^ Higher valencies increase statistical rebinding potential by increasing the effective local concentration of the multivalent binding molecules in the vicinity of the enzyme. Herein, we sought to explore the effect of homomultivalent TXA derivatives on plasmin inhibition via statistical rebinding. To achieve higher valencies, we utilized PAMAM (polyamidoamine) dendrimers. PAMAM dendrimers have been previously used in the synthesis of multivalent carbonic anhydrase (CA) inhibitors and were shown to improve inhibition with an increase in valency.^34^ PAMAM dendrimers of generation 0, 1, and 2 were used to synthesize multivalent TXA of valencies 4 (PAMAM^4^-TXA), 8 (PAMAM^8^-TXA), and 16 (PAMAM^16^-TXA), respectively. The synthesis of PAMAM^8^-TXA is shown in Scheme 2. All TXA dendrimers were synthesized by reacting PAMAM dendrimers with Fmoc-TXA in DMF at room temperature using HBTU, DIEA, and Oxyma Pure. The dendrimer product was precipitated with cold diethyl ether and washed with excess ether. The Fmoc was deprotected using 30% piperidine in DMF. The product was again precipitated with diethyl ether and washed with excess ether. This precipitate was solubilized in water and dialyzed using a Slide-A-Lyzer dialysis cassette (2 kDa MWCO) against deionized water to separate the byproducts from the conjugated dendrimer.^35^ In addition to these PAMAM^*X*^-TXA inhibitors, bivalent Bis-TXA was also synthesized using Fmoc-Lys­(Fmoc)-OH and Fmoc-TXA on a NovaPEG Rink amide resin via solid phase peptide synthesis (SPPS). The Fmoc was deprotected using 20% piperidine, and the compound was cleaved from the resin using 95% TFA/2.5% water/2.5% TIS (triisopropylsilane). All homomultivalent TXA molecules were finally purified using HPLC, and their masses were confirmed via a mass spectrometer, as described previously (see Figures S39–S42).

**Scheme 2:**
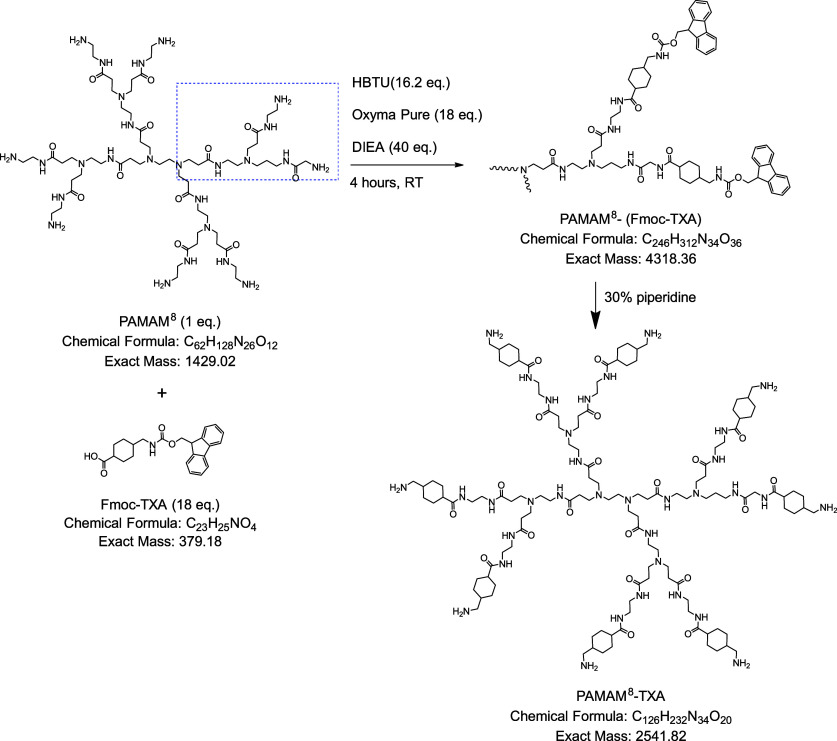
Synthesis Scheme for PAMAM^8^-TXA^a^

a PAMAM^8^-TXA was synthesized using PAMAM dendrimer (gen 2) and Fmoc-TXA using HBTU/DIEA/Oxyma Pure. Fmoc was deprotected using 30% piperidine to obtain PAMAM^8^-TXA.

### Plasmin Inhibition with Homomultivalent TXA Inhibitors

Monovalent TXA and Bis-TXA were found to be weak active site inhibitors of plasmin, with *K*_i_ values of 21 and 6.4 mM, respectively. This was expected as TXA predominantly binds to lysine binding sites (LBSs) on plasmin and is known to be a very weak active site inhibitor with a *K*_i_ of ∼40 mM reported in the literature.^36,37^ With an increase in valency from 1 to 8, homomultivalent TXA inhibitors became stronger active site inhibitors of plasmin. PAMAM^8^-TXA (*K*_i_ = 2.5 ± 1.8 μM) was the strongest inhibitor. It has a *K*_i_ value similar to that of pentamidine, the strongest multivalent benzamidine that was tested. The *K*_i_ value of PAMAM^16^-TXA was comparable to PAMAM^8^-TXA with a *K*_i_ value of 3.6 ± 3.0 μM. Therefore, increasing valency from 8 to 16 did not improve inhibition, and this is likely due to valency and size acting as neutralizing forces. An increase in valency increases the effective local concentration of the inhibitor moieties, and an increase in the size of the inhibitor decreases the effective local concentration. Size counteracted the effect of valency and did not improve overall inhibition. Hence, valencies beyond 16 were not tested. However, PAMAM^16^-TXA was a stronger inhibitor than other TXA inhibitors of valencies 1–4. The *K*_i_ values for all multivalent TXA inhibitors and monovalent TXA along with their separation lengths are shown in Table 3 (see Figures S43–49). PAMAM^8^-TXA, being the strongest homomultivalent TXA inhibitor, was tested for its inhibition of tPA and thrombin as well. PAMAM^8^-TXA exhibited stronger inhibition than monovalent TXA with *K*_i_ values of 43.3 ± 0.1 μM with tPA (rp 2,856, rp/*n* 357) and 15.5 ± 0.8 μM with thrombin (rp 13,573, rp/*n* 1,696). Monovalent TXA is a very weak active site inhibitor of tPA and thrombin with *K*_i_ values of 122,850 ± 33,950 and 210,382 ± 41,400 μM, respectively, indicating the potential enhancement in inhibition seen with PAMAM^8^-TXA due to secondary or nonspecific binding.

**Table 3:** Inhibition Constant (*K*_i_), rp, and rp/*n* Values for Multivalent Tranexamic Inhibitors with Plasmin along with the Theoretical Separation Lengths

inhibitors	*n*	*K*_i_ (μM)	rp^a^	rp/*n* ^b^	length (nm)^c^
TXA	1	21,370 ± 3300			1.0
Bis-TXA	2	6401 ± 2035	3.3	1.7	2.1
PAMAM^4^-TXA	4	183 ± 27	117	29	4.1
PAMAM^8^-TXA	8	2.5 ± 1.8	8548	1069	5.8
PAMAM^16^-TXA	16	3.6 ± 3.0	5936	371	7.6

a rp: relative potency = *K*_i_^TXA^/*K*_i_^multi^.

b rp/*n*: relative potency/number of TXA units

c Calculated planar separation lengths between TXA moieties measured end to end using ChemDraw (version 19.0.1.)

Monovalent TXA is known to inhibit fibrinolysis and not affect amidolysis.^33^ As S-2251 is a small, soluble tripeptide and is not representative of insoluble fibrin with multiple lysine residues, it can only determine the active site inhibition (amidolytic potential) of plasmin. Soluble substrate assays such as this cannot accurately determine a molecule’s overall fibrinolytic impact as it does not capture kringle–fibrin interactions.^22^ Therefore, it was interesting to observe lower *K*_i_ values and stronger inhibition with PAMAM^8^-TXA and PAMAM^16^-TXA. As S-2251 only measures active site inhibition by measuring the rate of substrate conversion by plasmin’s active site, these results indicate that TXA, a weak active site inhibitor that binds to the LBS on plasmin’s kringle domains, can be converted to a strong active site inhibitor employing multivalency.

Using TXA as a reference, the relative potency (rp) and relative potency per unit (rp/*n*) were calculated for homomultivalent TXA derivatives to determine the effect of multivalency on plasmin inhibition (Table 3). rp is the ratio of *K*_i_^TXA^ to *K*_i_^multi^ and is calculated to determine how potent the multivalent inhibitor is compared to its monovalent version. rp/*n* values were also calculated as they take into account valency and determine if linking multiple inhibitor moieties is beneficial. An rp/*n* > 1 indicates that linking multiple TXA moieties in a multivalent molecule is beneficial and each TXA moiety is more potent in a multivalent molecule compared to its monovalent version.^38^ All homomultivalent TXA inhibitors exhibited rp and rp/*n* values >1, indicating that the inhibitors portray multivalent effects and linking multiple TXA moieties together was beneficial. PAMAM^8^-TXA, the strongest inhibitor, displayed an rp value of 8548 indicating that it is 8548-fold more potent than monovalent TXA and an rp/*n* value of 1069 indicating that each TXA moiety in this molecule is 1069-fold more potent than monovalent TXA.

### Annular Fibrin Clot Assays

As S-2251 is a soluble substrate that measures only amidolytic potential, the true fibrinolytic potential of plasmin in the presence of LBS inhibitors such as TXA should be evaluated in physiologically relevant fibrin clots that can also capture fibrin–plasmin interactions. For this, we utilized fluorescently conjugated annular fibrin clots, which are insoluble and physiologically relevant. Annular clots utilize a fluorescently labeled fibrin clot that provides a unique annular geometry to monitor clot digestion kinetically in real time. They are fabricated in microplate wells using 3D printed inserts as described in Zeng et al.^39^ The annular clots are made from purified human thrombin (1 U/mL) and human fibrinogen (3 mg/mL with unmodified:∼12 FITC-tagged fibrinogen in a ratio of 50:1). The annular geometry allows for kinetic monitoring of clot lysis in real time by tracking the soluble FITC-tagged fibrin-degradation products released into the center of the well from insoluble fibrin as it is digested by plasmin. Inhibitory activity was assessed by calculating IC_50_ using *V*_max_ obtained at different inhibitor concentrations with 850 nM of plasmin. IC_50_ values were generated by three-parameter variable slope nonlinear regression using GraphPad Prism 9, version 9.2 (see Table 4 and Figures S50–S57).

**Table 4:** IC_50_ Values for Inhibitors with Annular Fibrin Clots Are Given along with *K*_i_/IC_50_ Values for Comparison

inhibitor	annular IC_50_ (μM)	IC_50_/*K*_i_
benzamidine	1141 ± 114	35.6
4-amino benzamidine	451 ± 70	8.8
4-aminomethyl benzamidine	32,673 ± 4318	29.0
pentamidine	3.2 ± 1.0	1.5
Tri-AMB	7.7 ± 3.0	2.0
EACA	4444 ± 473	0.06
TXA	896 ± 194	0.04
PAMAM^8^-TXA	64.8 ± 11.9	25.9

Comparing IC_50_ values from annular clot assays with *K*_i_ values from S-2251 assays, all inhibitors, except for TXA and EACA, exhibited higher IC_50_ values than *K*_i_ values. Pentamidine was found to be the strongest inhibitor of plasmin even in annular clot assays. Pentamidine and Tri-AMB had comparable IC_50_ values and exhibited similar *K*_i_ and IC_50_ values with IC_50_/*K*_i_ values of 1.5–2. In general, benzamidine derivatives followed similar inhibition trends but exhibited higher IC_50_ values in annular clots in comparison to *K*_i_ values. TXA and EACA, being LBS inhibitors, exhibited much lower IC_50_ values (stronger inhibition) with the annular clots compared to *K*_i_ values as expected, indicating that the annular fibrin clots take into account kringle binding and can capture the true fibrinolytic potential of plasmin in the presence of inhibitors. Interestingly, despite benzamidine being 660-fold stronger than TXA in the S-2251 assays, they exhibited comparable IC_50_ values. Benzamidine was found to be a slightly weaker inhibitor than TXA (IC_50_ 896 ± 194 μM) in annular clot assays with an IC_50_ value of 1,141 ± 114 μM, further signifying the importance of capturing kringle inhibition. Binding of TXA to the kringle domains of plasmin minimizes plasmin’s kringle binding to the fibrin and therefore reduces plasmin’s ability to digest the fibrin clot. Annular clot assays capture the inhibition of plasmin–fibrin interactions by TXA, whereas S-2251 does not. Therefore, *K*_i_ values derived from S-2251 assays are not always representative of the true fibrinolytic potential of nonactive site inhibitor molecules such as TXA and its derivatives. PAMAM^8^-TXA was found to be the strongest inhibitor of plasmin after pentamidine and Tri-AMB with an IC_50_ value of 64.8 ± 11.9 μM. As observed with the S-2251 assays, PAMAM^8^-TXA exhibited stronger (∼14-fold) inhibition than monovalent TXA in annular clots owing to the statistical rebinding effect of multivalency. These results demonstrate how the annular fibrin clot model can be utilized to evaluate the true fibrinolytic potential of diverse inhibitor molecules.

## Conclusions

Heterobivalent inhibitors and homomultivalent TXA derivatives were designed using principles of multivalency and structure-guided engineering. Strong plasmin inhibition can be achieved by leveraging the subsite binding effect of multivalency, as seen with bivalent inhibition by α2-antiplasmin *in vivo*. For this, an appropriate linker length that allows for simultaneous binding to both the active site and LBS on plasmin kringle domains is necessary. Heterobivalent inhibitors of various linker lengths from ∼4 to 17 nm were synthesized, and it was observed that TXA-dPEG_4_-AMB was the strongest heterobivalent inhibitor for plasmin with a *K*_i_ value of 42 ± 12 μM. Increasing linker lengths from dPEG_4_ to dPEG_12_, which are shorter than the distance between the active site and LBS on kringle domains, resulted in weaker inhibition as seen previously with multivalent benzamidines of long linker lengths.^28^ However, despite its long linker length, dPEG_36_ exhibited improved inhibition of plasmin with a *K*_i_ value of 75 ± 4.3 μM. TXA-dPEG_36_-AMB (∼17 nm) allowed benzamidine to bind to the active site and TXA to bind to the LBS on K1 of plasmin simultaneously and resulted in improved inhibition through multivalent subsite binding. Even with tPA, subsite biding effects were potentially achieved as TXA-dPEG_36_-AMB was found to be the strongest heterobivalent inhibitor of tPA with a *K*_i_ value of 136 ± 0.7 μM. As thrombin does not have any kringle domains, inhibition steadily weakened with longer linker lengths. The *K*_i_ values of TXA-dPEG*x*-AMB inhibitors are <17-fold different across all three serine proteases, i.e, plasmin, tPA, and thrombin, indicating potential issues in selectivity. The linkers are further being optimized to achieve selectivity toward a specific serine protease. Once more selective candidates are identified, these heteromultivalent inhibitors will be tested in multienzyme and substrate systems and blood flow models. We also plan to use covalent and irreversible inhibitors of plasmin, such as α2-antiplasmin and macroglobulin, to study their effect on multivalent inhibitors once more selective inhibitors are identified.

Regarding homomultivalent TXA derivatives, it was determined that TXA, which is a weak active site inhibitor of plasmin with a *K*_i_ value of 21,370 ± 3300 μM, can be transformed into a strong active site inhibitor utilizing a statistical rebinding mechanism of multivalency. It was observed that plasmin inhibition by PAMAM^*X*^-TXA dendrimers became stronger with increasing valency until PAMAM^8^-TXA, which was the strongest with *K*_i_ value of 2.5 ± 1.8 μM. At valencies above PAMAM^8^-TXA, such as PAMAM^16^-TXA, decreased inhibition was observed likely due to the size of the molecule counteracting the increased effective concentration of TXA at higher valency. PAMAM^16^-TXA was still a stronger inhibitor than homomultivalent TXA derivatives of valencies 1–4. Therefore, multivalency is a unique and highly effective strategy that can be leveraged to design enzyme inhibitors. To capture kringle interactions and determine the fibrinolytic potential of plasmin rather than just its amidolytic potential in the presence of inhibitors, we also performed experiments with physiologically relevant, fluorescently tagged annular fibrin clots. The IC_50_ values obtained were compared with *K*_i_ values determined using soluble substrate enzyme inhibition assays. Benzamidine derivatives had larger IC_50_ values but followed similar inhibition trends, as seen with the soluble substrate enzyme inhibition assays. Multivalent pentamidine and Tri-AMB were found to be stronger inhibitors of plasmin than monovalent benzamidine derivatives, as expected. However, LBS inhibitors such as TXA and EACA were found to be much stronger inhibitors (lower IC_50_ values) in the annular clot assays, indicating that the annular clot assay can capture the inhibition of kringle domains by these LBS inhibitors that S-2251 could not. PAMAM^8^-TXA was also found to be a stronger inhibitor (lower IC_50_ value) than monovalent TXA in the annular fibrin clot assay as well. Therefore, when evaluating purely LBS inhibitors, it is important to use physiologically relevant fibrin clot models that can capture kringle–fibrin interactions to evaluate the true fibrinolytic impact on plasmin in the presence of inhibitors.

## Methods

### Synthesis and Purification of Inhibitors

All heterobivalent inhibitors were synthesized utilizing AMB, TXA (or EACA), and Fmoc-dPEG*x*-NHS/TFP esters. AMB was first reacted with Fmoc-dPEG*x*-NHS/TFP esters (*x* = 4, 8, 12, and 36), and then Fmoc was deprotected using 30% piperidine in DMF to yield NH_2_-dPEG*x*-AMB. This was purified on HPLC and then reacted with Fmoc-TXA (or Fmoc-EACA) using 2-(1*H*-benzotriazol-1-yl)-1,1,3,3-tetramethyluronium hexafluorophosphate (HBTU) and *N*,*N*-diisopropylethylamine (DIEA). Fmoc was again deprotected with 30% piperidine. The final product was purified on a Thermo HPLC (high-performance liquid chromatography) using a semipreparative Thermo Hypersil GOLD C18 column (5 μm, 250 × 10 mm) on a gradient of water and methanol with 0.1% trifluoroacetic acid (TFA). The masses were confirmed using an Agilent LC1290 Infinity II MS6545 Q-ToF system in positive ion mode at a fragmentation voltage of 220 V.

Homomultivalent PAMAM dendrimers of generation 0 to 2 corresponding to valencies of 4 to 16 were synthesized using PAMAM dendrimers and Fmoc-TXA using HBTU, DIEA, and Oxyma Pure. The Fmoc was deprotected using 30% piperidine in DMF. The product was first precipitated with diethyl ether and washed with excess ether. It was then solubilized in water and was dialyzed using a Slide-A-Lyzer dialysis cassette (2 kDa MWCO) against deionized water to separate out the byproducts. Bis-TXA was synthesized by using Fmoc-Lys­(Fmoc)-OH and Fmoc-TXA on a NovaPEG Rink amide resin via solid phase peptide synthesis (SPPS). The Fmoc was deprotected using 20% piperidine, and the compound was cleaved from the resin using 95% TFA/2.5% TIS (triisopropylsilane)/2.5% water. All molecules were HPLC purified and mass spectrometry verified as detailed above.

### Inhibition Assays

Inhibition assays with plasmin, tPA, and thrombin were performed using chromogenic and fluorogenic substrates S-2251 (Chromogenix, H-D-Val-Leu-Lys-pNA•2HCl), S-2288 (H-D-lle-Pro-Arg-pNA•2HCl), and Thrombin Substrate III (TSIII; Benzoyl-Phe-Val-Arg-AMC•HCl), respectively. Plasmin inhibition assays were carried out at a fixed concentration of human plasmin (42.5 nM) over a range of inhibitor concentrations (0–300,000 μM) and S-2251 concentrations (100–500 μM). Plasmin activity was determined using initial velocities (Vo) in μM/min that were calculated for each inhibitor and substrate concentration measuring the slope of release of *p*-nitroaniline by hydrolysis of S-2251 by plasmin in the presence of the inhibitor at 405 nm. *K*_i_ values were determined using Vo by calculating the *x*-axis value of the negative intersection point utilizing Dixon plot analysis. Cornish–Bowden graphs (*S*/Vo vs *I*) were also plotted to determine if the inhibition was competitive, uncompetitive, or noncompetitive. Similarly, inhibition assays for tPA were carried out using a range of S-2288 concentrations (100–500 μM) and inhibitor concentrations (0–300,000 μM) and a fixed concentration of human tPA (75 nM) tracking at 405 nm. Inhibition assays for thrombin utilized fluorogenic TSIII concentrations of 20–50 μM, inhibitor concentrations of 0–300,000 μM, and a fixed human thrombin concentration of 0.25 U/mL. Thrombin activity was determined by monitoring the fluorescent AMC tag (λex: 370 nm; λem: 450 nm) released by the hydrolysis of TSIII.

### Annular Clot Fabrication and IC_50_ Assays

Annular clots were fabricated using a 3D printed insert into a 96-plate well having 80 μL of clotting solution as described in Zeng et al.^39^ The clotting solution contained purified human fibrinogen at final concentrations of 3 mg/mL fibrinogen (having 50:1 unmodified fibrinogen/FITC-tagged fibrinogen) and 1 U/mL thrombin. The insert was carefully removed after 30 min of clotting, and the annular clots were gently washed with 0.01 M PBS twice and stored in 120 μL of PBS before use. For all IC_50_ experiments, a 120 μL sample solution comprising 850 nM plasmin incubated with different concentrations of inhibitors ranging from 0 to 1,000,000 μM was added to the center of the annular clot to initiate clot lysis. Each inhibitor concentration was run in triplicates. Fluorescence (Ex 495, Em 519) was monitored for 60 min reading every 30 s, and clot lysis was determined by calculating *V*_max_, the maximum rate of fluorescence. Inhibitory activity was assessed by calculating IC_50_ using *V*_max_ obtained at different inhibitor concentrations. IC_50_ values were generated by three-parameter variable slope nonlinear regression using GraphPad Prism 9, version 9.2.

## Supplementary Material



## Notes

Additional synthesis details, ChemDraw structures, HPLC traces, *m*/*z* values, Dixon and Cornish–Bowden plots, and IC_50_ curves for all inhibitors (PDF)

## ABBREVIATIONS

AMB: 4-aminomethyl benzamidine
EACA: ε-aminocaproic acid
HPLC: high-performance liquid chromatography
LBS: lysine binding site
MS: mass spectrometry
PAMAM: polyamidoamine
PEG: polyethylene glycol
TFA: trifluoroacetic acid
tPA: tissue plasminogen activator
TSIII: thrombin substrate III
TXA: tranexamic acid
