# Supplementary material for: Multivalent Tranexamic Acid (TXA) and Benzamidine Derivatives for Serine Protease Inhibition
Source: ACS Pharmacol Transl Sci. 2025 May 15;8(6):1669–77. doi: 10.1021/acsptsci.5c00030 (PMC12186753; doi:10.1021/acsptsci.5c00030)
Supplement: Supplementary file 1 [file pt5c00030_si_001.pdf]

Supporting Information

Multivalent Tranexamic Acid (TXA) and Benzamidine Derivatives for Serine Protease Inhibition

Tanmaye Nallan Chakravarthula [a,b], Rodrigo Santillan-Rodriguez [a], Ziqian Zeng [a,b], Abigail Hall [a], Andres Prieto Trujillo [a], Anushri Umesh [a,b], Nathan J. Alves [a, b] *
[a] Department of Emergency Medicine, Indiana University School of Medicine, Indiana University, Indianapolis, IN 46202, USA
[b] Weldon School of Biomedical Engineering, Purdue University, West Lafayette, IN 47907, USA
*Corresponding author (nalves@iu.edu)

Table of Contents

S1 General Experimental Procedures……………………………………………………………………………….........4

S2 Inhibition with Benzamidine Derivatives………………………………………..………………..…………………5

S2.1 tPA inhibition with benzamidine derivatives

Figure S1: K_i determination of benzamidine

Figure S2: K_i determination of 4-amino benzamidine

Figure S3: K_i determination of 4-aminomethyl benzamidine

Figure S4: K_i determination of pentamidine

Figure S5: K_i determination of Tri-AMB

S2.2 Thrombin inhibition with benzamidine derivatives

Figure S6: K_i determination of benzamidine

Figure S7: K_i determination of 4-amino benzamidine

Figure S8: K_i determination of 4-carboxy benzamidine

Figure S9: K_i determination of 4-aminomethyl benzamidine

Figure S10: K_i determination of pentamidine

Figure S11: K_i determination of Tri-AMB

S3 Synthesis and Characterization of Hetero-bivalent Inhibitors…………………………………..........................10

Figure S12: Chemical structure and characterization of TXA-dPEG$_4$-AMB

Figure S13: Chemical structure and characterization of TXA-dPEG$_8$-AMB

Figure S14: Chemical structure and characterization of TXA-dPEG$_{12}$-AMB

Figure S15: Chemical structure and characterization of TXA-dPEG$_{36}$-AMB

Figure S16: Chemical structure and characterization of EACA-dPEG$_4$-AMB

S4 Inhibition Assays with Hetero-bivalent Inhibitors…………………………….……………………………………...13

S4.1 Plasmin inhibition with hetero-bivalent inhibitors

Figure S17: K_i determination of TXA

Figure S18: K_i determination of TXA-dPEG$_4$-AMB

Figure S19: K_i determination of TXA-dPEG$_8$-AMB

Figure S20: K_i determination of TXA-dPEG$_{12}$-AMB

Figure S21: K_i determination of TXA-dPEG$_{36}$-AMB

Figure S22: K_i determination of EACA

Figure S23: K_i determination of EACA-dPEG$_4$-AMB

Figure S24: Chimera figure of plasmin and TXA-dPEG$_{36}$-AMB

S4.2 tPA inhibition with hetero-bivalent inhibitors

Figure S25: K_i determination of TXA

Figure S26: K_i determination of TXA-dPEG$_4$-AMB

Figure S27: K_i determination of TXA-dPEG$_8$-AMB

Figure S28: K_i determination of TXA-dPEG$_{12}$-AMB

Figure S29: K_i determination of TXA-dPEG$_{36}$-AMB

Figure S30: K_i determination of EACA

Figure S31: K_i determination of EACA-dPEG$_4$-AMB

S4.3 Thrombin inhibition with hetero-bivalent inhibitors

Figure S32: K_i determination of TXA

Figure S33: K_i determination of TXA-dPEG$_4$-AMB

Figure S34: K_i determination of TXA-dPEG$_8$-AMB

Figure S35: K_i determination of TXA-dPEG$_{12}$-AMB

Figure S36: K_i determination of TXA-dPEG$_{36}$-AMB

Figure S37: K_i determination of EACA

Figure S38: K_i determination of EACA-dPEG$_4$-AMB

S5 Synthesis and Characterization of Homo-multivalent TXA Inhibitors …..……………..…….…………………...24

Figure S39: Chemical structure and characterization of Bis-TXA

Figure S40: Chemical structure and characterization of PAMAM^4-TXA

Figure S41: Chemical structure and characterization of PAMAM^8-TXA

Figure S42: Chemical structure and characterization of PAMAM^{16}-TXA

S6 Inhibition Assays for Homo-multivalent TXA Inhibitors………………………….…………………......................27

Figure S43: K_i determination of TXA with plasmin

Figure S44: K_i determination of Bis-TXA with plasmin

Figure S45: K_i determination of PAMAM^4-TXA with plasmin

Figure S46: K_i determination of PAMAM^8-TXA with plasmin

Figure S47: K_i determination of PAMAM^8-TXA with tPA

Figure S48: K_i determination of PAMAM^8-TXA with thrombin

Figure S49: K_i determination of PAMAM^{16}-TXA with plasmin

S7 Annular Fibrin Clot Assays…………………………………………………………….……….……..........................30

Figure S50: IC_{50} with benzamidine

Figure S51: IC_{50} with 4-amino benzamidine

Figure S52: IC_{50} with 4-aminomethyl benzamidine

Figure S53: IC_{50} with Pentamidine

Figure S54: IC_{50} with Tri-AMB

Figure S55: IC_{50} with EACA

Figure S56: IC_{50} with TXA

Figure S57: IC_{50} with PAMAM^8-TXA

S8 Table………………………………………………………….…………...33

Table S1

S1 General Experimental Procedures

Synthesis of inhibitors: Hetero-bivalent inhibitors of different dPEG lengths were synthesized utilizing AMB, TXA (or EACA) and Fmoc-dPEG$_x$-NHS/TFP esters. AMB was first reacted with Fmoc-dPEG$_x$-NHS/TFP esters (x = 4,8,12,36) in a mixture of DMF and PBS. The reaction crude was dried and the Fmoc was deprotected using 30% piperidine in DMF. The reaction crude was dried again and the product NH_2-dPEG$_x$-AMB was selectively solubilized in water and then was purified on HPLC using semi-preparative Thermo Hypersil GOLD C18 column (5µm, 250 x 10mm) on a gradient of water and methanol with 0.1% trifluoroacetic acid (TFA). The masses were confirmed using mass spectrometry on Agilent LC1290 Infinity II MS6545 Q-ToF system in positive ion mode at a fragmentation voltage of 220 V. The NH_2-dPEG$_x$-AMB were then reacted with Fmoc-TXA (or Fmoc-EACA) in DMF at room temperature using 2-(1H-benzotriazol-1-yl)-1,1,3,3-tetramethyluronium hexafluorophosphate (HBTU) and N,N-Diisopropylethylamine (DIEA). Finally, Fmoc was deprotected using 30% piperidine and the product was purified on HPLC and confirmed with mass spectrometry. Masses obtained were compared with the exact masses that were determined from the structures drawn with ChemDraw (Version 19.0.1.28).

PAMAM dendrimers of generation 0 to 2 corresponding to valencies of 4 to 16 were used to synthesize multivalent TXA of valencies 4 (PAMAM^4-TXA), 8 (PAMAM^8–TXA) and 16 (PAMAM^{16}–TXA). These dendrimers were synthesized using PAMAM dendrimers and Fmoc-TXA in DMF at room temperature using HBTU, DIEA and Oxyma Pure. The dendrimer product was precipitated with cold ethyl ether and washed with excess ether. The Fmoc was deprotected using 30% piperidine in DMF. This was again precipitated with diethyl ether and washed with excess ether. This precipitate was solubilized in water and was dialyzed using Slide-A-Lyzer dialysis cassette against deionized water having 2 kDa MWCO to separate out the by-products from the conjugated dendrimer. Finally, the product of intended valency was purified using HPLC semi-preparative Thermo Hypersil GOLD C18 column (5µm, 250 x 10mm) on a gradient of water and methanol with 0.1% TFA and the masses were confirmed using mass spectrometry. In addition to these dendrimer-TXA inhibitors, bivalent Bis-TXA was also synthesized using Fmoc-Lys(Fmoc)-OH and Fmoc-TXA on a NovaPEG Rink amide resin via solid phase peptide synthesis (SPPS). The Fmoc was deprotected using 20% piperidine and the compound was cleaved from the resin using 95%TFA/ 2.5%TIS(Triisopropylsilane)/ 2.5% water. This was purified on HPLC using the method mentioned above and the mass was verified via mass spectrometry.

Inhibition Assays: Inhibition assays with plasmin, tpa and thrombin were performed to determine and compare inhibition constants (K_i) using Dixon plot analysis. Chromogenix S-2251(H-D-Val-Leu-Lys-pna•2hcl), a chromogenic substrate for plasmin was used to determine inhibition constants (K_i) with plasmin. For each inhibitor, an inhibition assay was carried out at a fixed concentration of human plasmin (42.5 nm) over a range of inhibitor concentrations (0-300,000 µm) and S-2251 concentrations (100-500 µm). Plasmin activity was determined using initial velocities (V_o) in µm/min which were calculated for each inhibitor and substrate concentration by calculating the slope of release of p-Nitroaniline by hydrolysis of S-2251 by plasmin in presence of inhibitor at 405 nm. V_o values were determined in a Corning™ 96-Well Nonbinding Surface (NBS™) Microplates (3641) using Molecular Devices spectramax® M5 Microplate Reader. K_i values were calculated using the V_o values obtained for each inhibitor at different inhibitor and substrate concentrations by calculating the x-axis value of the negative intersection point in Dixon plot analysis. Cornish-Bowden graphs (S/Vo vs I) were also plotted to determine if the inhibition was competitive, uncompetitive, or non-competitive. Similarly, inhibition assays for tpa were carried out using Chromogenix S-2288 (H-D-Ile-Pro-Arg-pna•2hcl), a chromogenic substrate for tpa. A range of S-2288 concentrations (100-500 µm), inhibitor concentrations (0-300,000 µm), and a fixed concentration of human tpa (75 nm) were utilized to perform inhibition assays with tpa. tpa activity was also tracked at 405 nm by measuring hydrolysis of S-2288. Inhibition assays for thrombin utilized fluorogenic Thrombin Substrate III (TSIII; Benzoyl-Phe-Val-Arg-AMC•hcl) and were carried out at TSIII concentrations of 20-50 µm, inhibitor concentrations of 0-300,000 µm, and a fixed human thrombin concentration of 0.25 U/ml. Thrombin activity was determined by monitoring the fluorescent AMC tag (λex: 370 nm and λem: 450 nm) released by hydrolysis of TSIII.

Annular Clot Fabrication and IC_{50} assays: Annular clots were fabricated using a 3D printed insert into a 96-plate well having 80 µL of clotting solution. The clotting solution comprises of purified human fibrinogen and human thrombin at final concentrations of 3 mg/mL Fbg and 1U/mL thrombin respectively. To make the annular clots, 5 µL of 16U/mL thrombin was added to 75 µL of 3.2 mg/mL fibrinogen (having 50:1 of unmodified fibrinogen: FITC-tagged fibrinogen) and the 3D printed insert was inserted into this clotting solution. After 30 mins of clotting at room temperature, the insert was carefully removed, and the annular clots were gently washed with 0.01 M PBS twice and stored in 120 µL PBS before use. For all IC_{50}

experiments, a 120 µL sample solution comprising 850 nM plasmin incubated with different concentrations of inhibitors ranging from 0-1,000,000 µM was added to the center of the annular clot to initiate clot lysis. Each inhibitor concentration was run in triplicates. Fluorescence (Ex 495, Em 519) was monitored for 60 min reading every 30 seconds and clot lysis was determined by calculating V_{max}, the maximum rate of fluorescence. Inhibitory activity was assessed by calculating IC_{50} using V_{max} obtained at different inhibitor concentrations. IC_{50} values were generated by three-parameter variable slope non-linear regression, using GraphPad Prism 9, version 9.2.

S2 Inhibition with benzamidine derivatives

S2.1 tPA inhibition with benzamidine derivatives

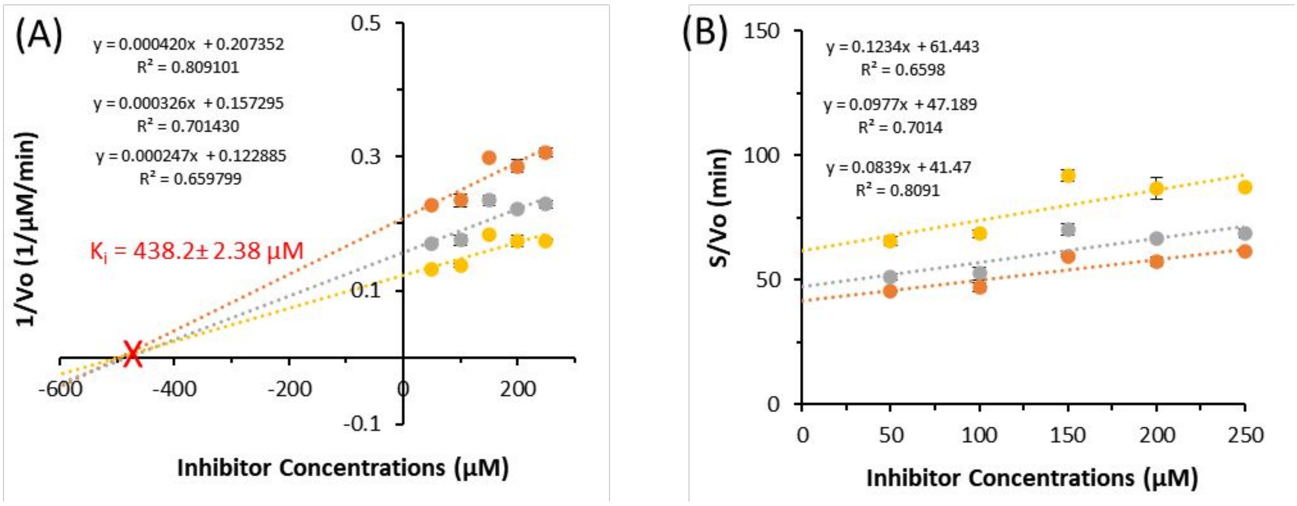

Figure S1: (A) K_i determination of benzamidine utilizing a Dixon Plot. 0 – 250 µM of benzamidine was incubated with a fixed tPA concentration of 75 nM in PBS pH 7.4. Three different S-2258 concentrations of 200 µM (orange), 300 µM (gray) and 500 µM (yellow) were utilized to obtain K_i which is the negative intersection of the lines at 438.2 ± 2.38 µM. (B) Cornish-Bowden S/Vo vs I plot was used to determine the mode of inhibition. Benzamidine was found to be a competitive inhibitor as the lines in this plot are parallel. All data is represented as mean ± SD of triplicate experiments.

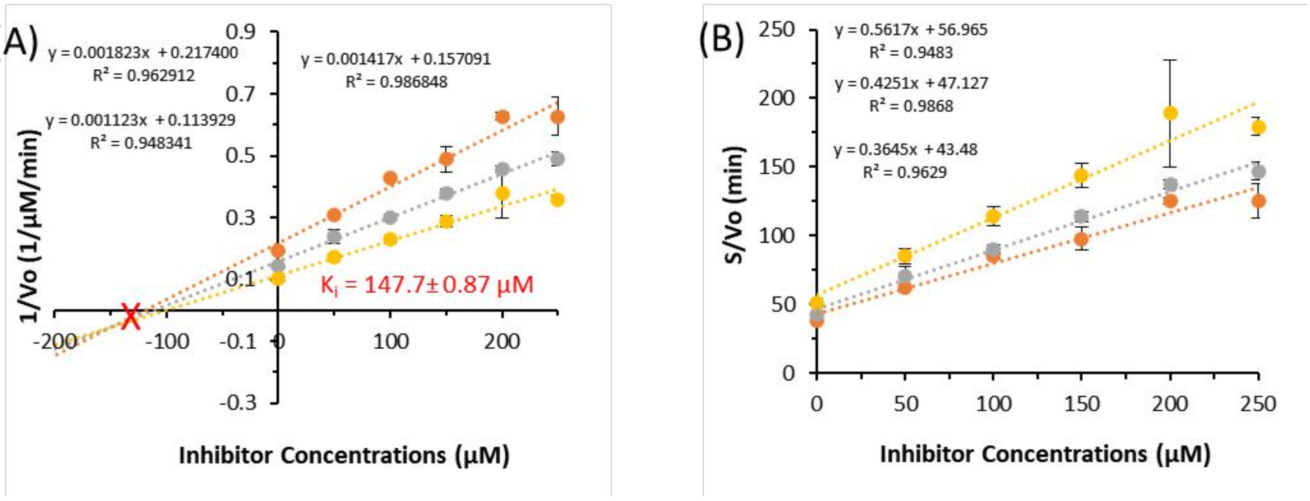

Figure S2: (A) K_i determination of 4-amino benzamidine utilizing a Dixon Plot. 0 – 250 µM of 4-amino benzamidine was incubated with a fixed tPA concentration of 75 nM in PBS pH 7.4. Three different S-2258 concentrations of 200 µM (orange), 300 µM (gray) and 500 µM (yellow) were utilized to obtain K_i which is the negative intersection of the lines at 147.7 ± 0.87 µM. (B) Cornish-Bowden S/Vo vs I plot was used to determine the mode of inhibition. 4-amino benzamidine was found to be a competitive inhibitor as the lines in this plot are parallel. All data is represented as mean ± SD of triplicate experiments.

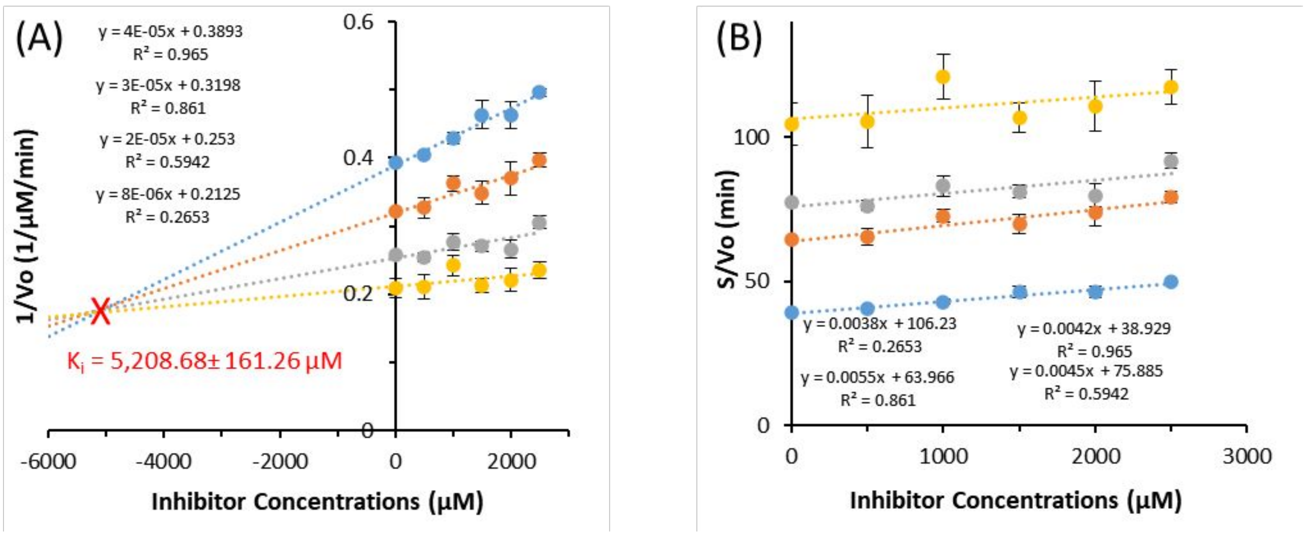

Figure S3: (A) K_i determination of 4-aminomethyl benzamidine utilizing a Dixon Plot. 0 – 2,500 µM of 4-aminomethyl benzamidine was incubated with a fixed tPA concentration of 75 nM in PBS pH 7.4. Four different S-2258 concentrations of 150 µM (blue), 200 µM (orange), 300 µM (gray) and 500 µM (yellow) were utilized to obtain K_i which is the negative intersection of the lines at 5,208.68 ± 161.26 µM. (B) Cornish-Bowden S/Vo vs I plot was used to determine the mode of inhibition. 4-aminomethyl benzamidine was found to be a competitive inhibitor as the lines in this plot are parallel. All data is represented as mean ± SD of triplicate experiments.

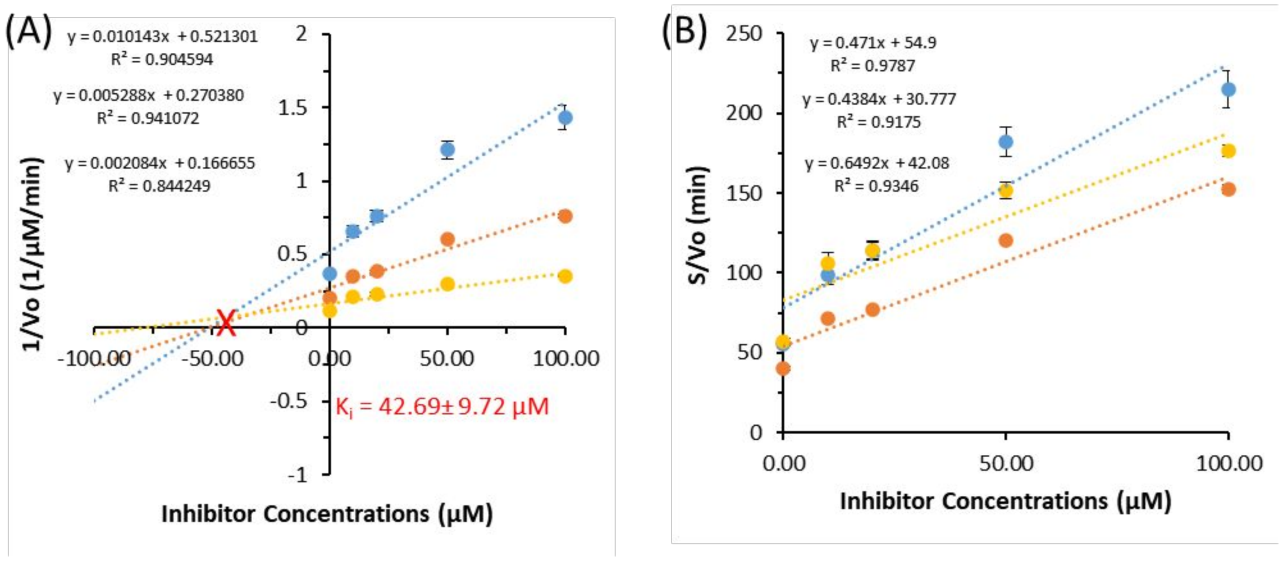

Figure S4: (A) K_i determination of pentamidine utilizing a Dixon Plot. 0 – 100 µM of pentamidine was incubated with a fixed tPA concentration of 75 nM in PBS pH 7.4. Three different S-2258 concentrations of 100 µM (blue), 200 µM (orange), and 500 µM (yellow) were utilized to obtain K_i which is the negative intersection of the lines at 42.69 ± 9.72 µM. (B) Cornish-Bowden S/Vo vs I plot was used to determine the mode of inhibition. Pentamidine was found to be a competitive inhibitor. All data is represented as mean ± SD of triplicate experiments.

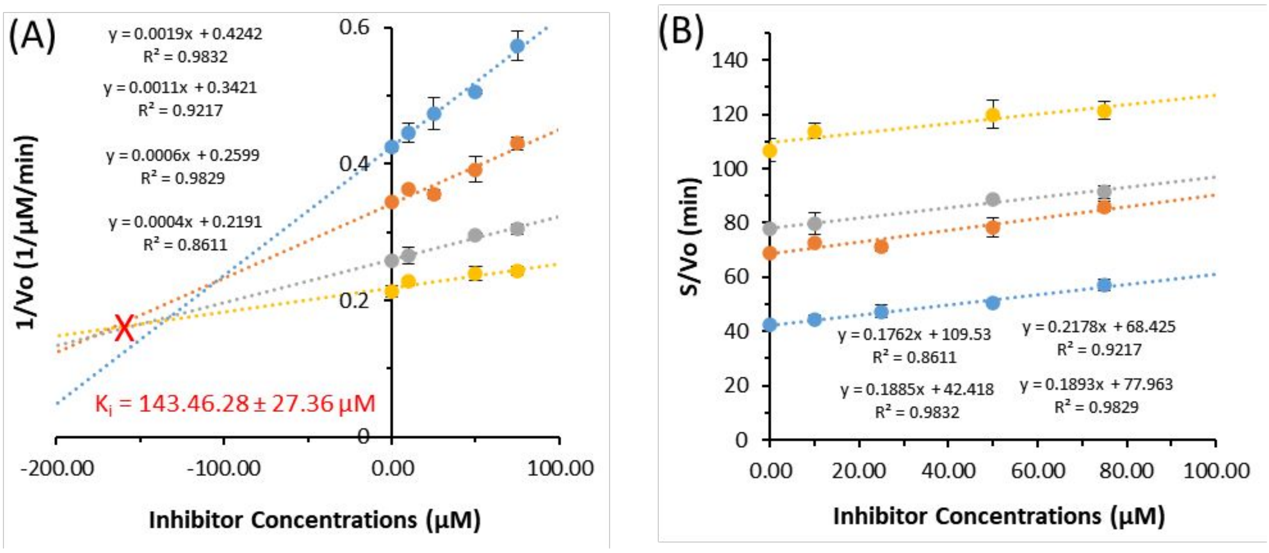

Figure S5: (A) K_i determination of Tri-AMB utilizing a Dixon Plot. 0 – 75 µM of Tri-AMB was incubated with a fixed tPA concentration of 75 nM in PBS pH 7.4. Four different S-2258 concentrations of 150 µM (blue), 200 µM (orange), 300 µM (gray) and 500 µM (yellow) were utilized to obtain K_i which is the negative intersection of the lines at 143.46 ± 27.36 µM. (B) Cornish-Bowden S/Vo vs I plot was used to determine the mode of inhibition. Tri-AMB was found to be a competitive inhibitor as the lines in this plot are parallel. All data is represented as mean ± SD of triplicate experiments.

S2.2 Thrombin inhibition with benzamidine derivatives

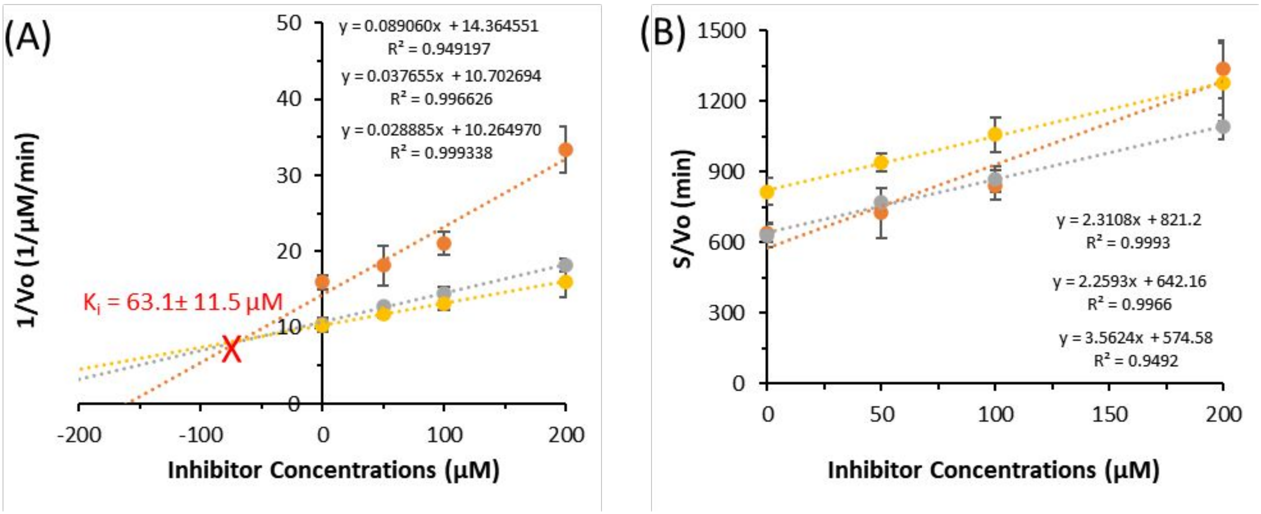

Figure S6: (A) K_i determination of benzamidine utilizing a Dixon Plot. 0 – 200 µM of benzamidine was incubated with a fixed thrombin concentration of 0.25 U/mL in PBS pH 7.4 at 10% DMSO. Three different TSIII concentrations of 40 µM (orange), 60 µM (gray), and 80 µM (yellow) were utilized to obtain K_i which is the negative intersection of the lines at 63.1 ± 11.5 µM. (B) Cornish-Bowden S/Vo vs I plot was used to determine the mode of inhibition. Benzamidine was found to be a competitive inhibitor. All data is represented as mean ± SD of triplicate experiments.

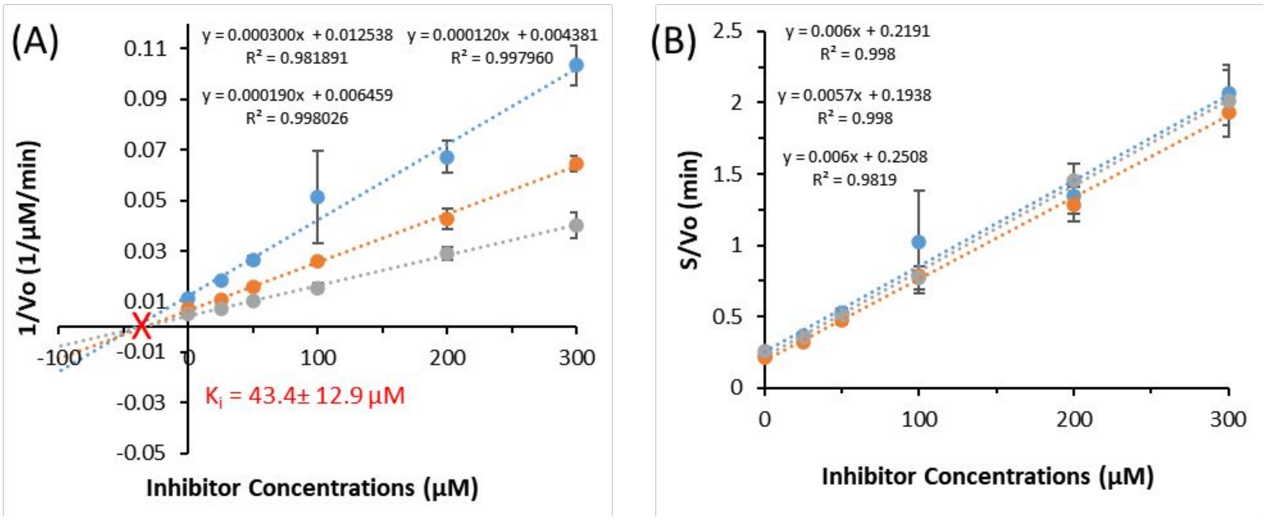

Figure S7: (A) K_i determination of 4-amino benzamidine utilizing a Dixon Plot. 0 – 300 µM of 4-amino benzamidine was incubated with a fixed thrombin concentration of 0.25 U/mL in PBS pH 7.4 at 10% DMSO. Three different TSIII concentrations of 20 µM (blue), 30 µM (orange), and 50 µM (gray) were utilized to obtain K_i which is the negative intersection of the lines at 43.4 ± 12.9 µM. (B) Cornish-Bowden S/Vo vs I plot was used to determine the mode of inhibition. 4-amino benzamidine was found to be a competitive inhibitor as the lines in this plot are parallel. All data is represented as mean ± SD of triplicate experiments.

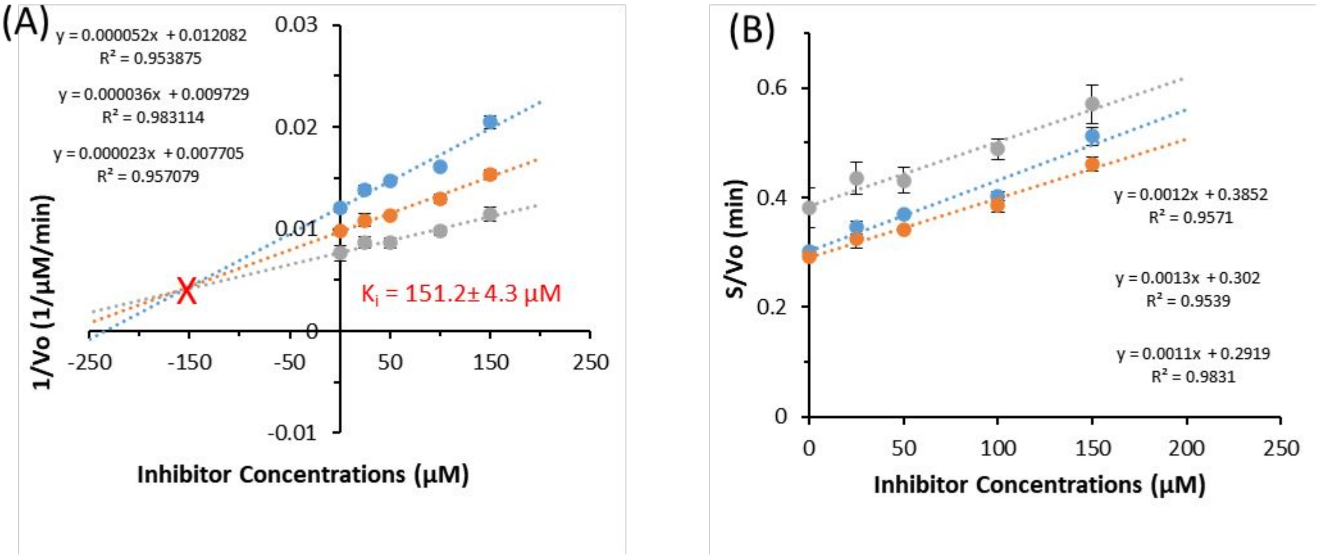

Figure S8: (A) K_i determination of 4-carboxy benzamidine utilizing a Dixon Plot. 0 – 150 µM of 4-carboxy benzamidine was incubated with a fixed thrombin concentration of 0.25 U/mL in PBS pH 7.4 at 10% DMSO. Three different TSIII concentrations of 25 µM (blue), 30 µM (orange), and 50 µM (gray) were utilized to obtain K_i which is the negative intersection of the lines at 151.2 ± 4.3 µM. (B) Cornish-Bowden S/Vo vs I plot was used to determine the mode of inhibition. 4-carboxy benzamidine was found to be a competitive inhibitor as the lines in this plot are parallel. All data is represented as mean ± SD of triplicate experiments.

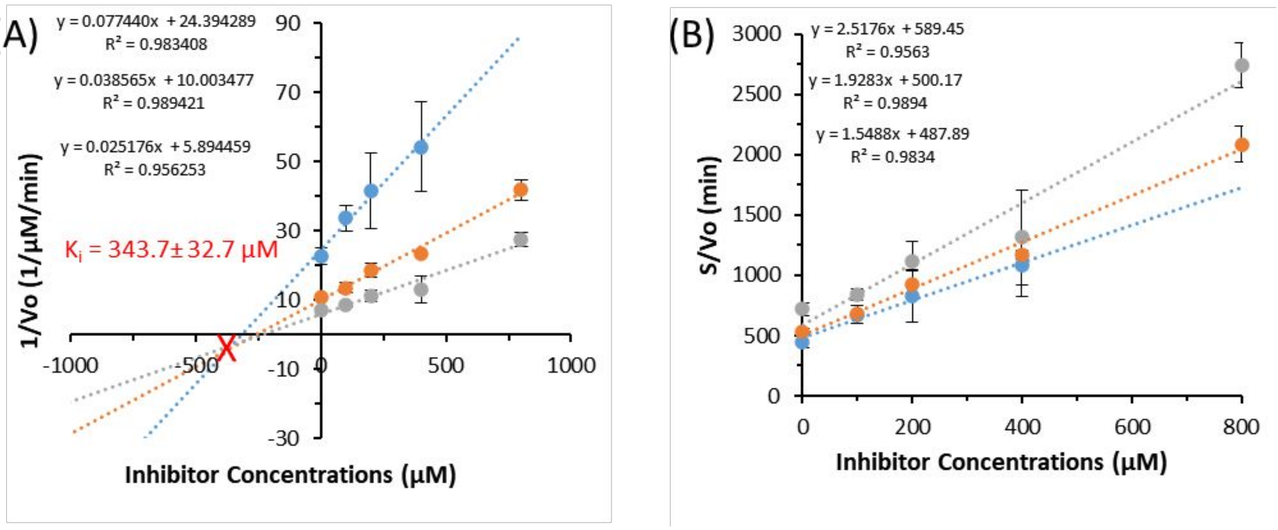

Figure S9: (A) K_i determination of 4-aminomethyl benzamidine utilizing a Dixon Plot. 0 – 800 μM of 4-aminomethyl benzamidine was incubated with a fixed thrombin concentration of 0.25 U/mL in PBS pH 7.4 at 10% DMSO. Three different TSIII concentrations of 20 μM (blue), 50 μM (orange), and 100 μM (gray) were utilized to obtain K_i which is the negative intersection of the lines at 343.7 ± 32.7 μM. (B) Cornish-Bowden S/Vo vs I plot was used to determine the mode of inhibition. 4-aminomethyl benzamidine was found to be a competitive inhibitor as the lines in this plot are parallel. All data is represented as mean ± SD of triplicate experiments.

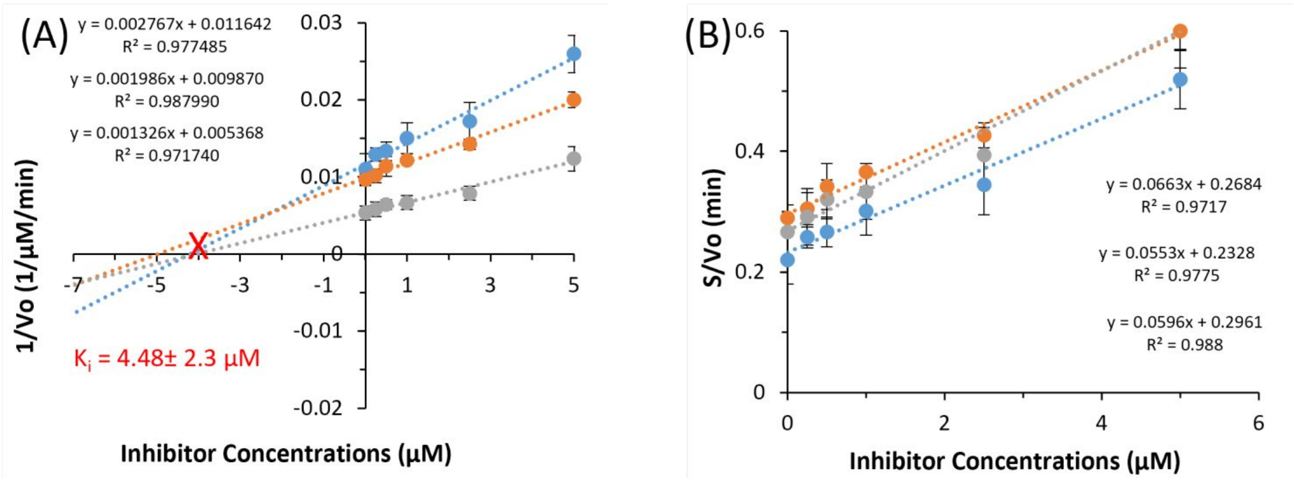

Figure S10: (A) K_i determination of pentamidine utilizing a Dixon Plot. 0 – 5 μM of pentamidine was incubated with a fixed thrombin concentration of 0.25 U/mL in PBS pH 7.4 at 10% DMSO. Three different TSIII concentrations of 20 μM (blue), 30 μM (orange), and 50 μM (gray) were utilized to obtain K_i which is the negative intersection of the lines at 4.48 ± 2.3 μM. (B) Cornish-Bowden S/Vo vs I plot was used to determine the mode of inhibition. Pentamidine was found to be a competitive inhibitor as the lines in this plot are parallel. All data is represented as mean ± SD of triplicate experiments.

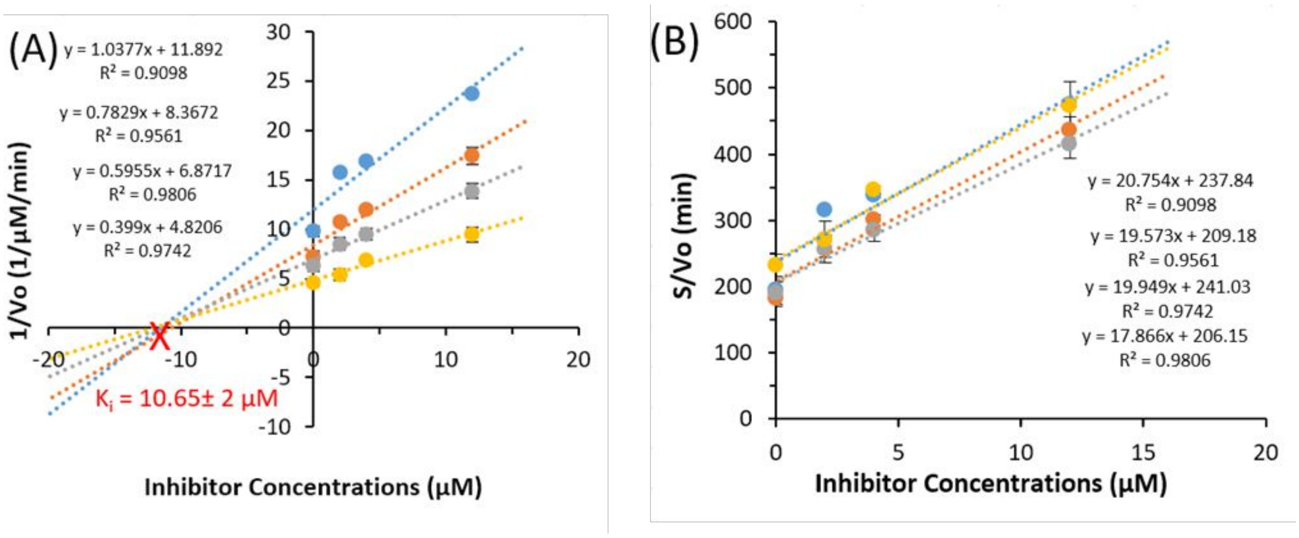

Figure S11: (A) K_i determination of Tri-AMB utilizing a Dixon Plot. 0 – 12 μM of Tri-AMB was incubated with a fixed thrombin concentration of 0.25 u/mL in PBS pH 7.4 at 10% DMSO. Four different TSIII concentrations of 20 μM (blue), 25 μM (orange), 30 μM (gray) and 50 μM (yellow) were utilized to obtain K_i which is the negative intersection of the lines at 10.65 ± 2 μM. (B) Cornish-Bowden S/Vo vs I plot was used to determine the mode of inhibition. Tri-AMB was found to be a competitive inhibitor as the lines in this plot are parallel. All data is represented as mean ± SD of triplicate experiments.

S3 Synthesis and Characterization of Hetero-bivalent Inhibitors

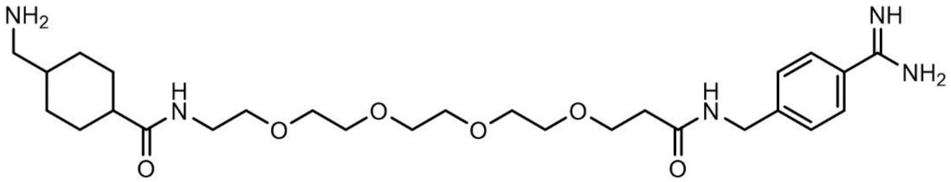

TXA-PEG4-AMB
Chemical Formula: $C_{27}H_{45}N_5O_6$
Exact Mass: 535.34

Figure S12: Chemical Structure and characterization of TXA-dPEG$_4$-AMB. To synthesize TXA-dPEG$_4$-AMB, NH_2-dPEG$_4$-AMB was first synthesized by reacting Fmoc-N-amido-dPEG$_4$-NHS (1 eq., 40 μmol) with AMB (3.25 eq., 130 μmol) in a mixture of 70% DMF and 30% 0.01M PBS at pH 7.4. 15uL of Triethylamine(TEA) was added and the sample was stirred for 30 mins at room temperature. Fmoc was then deprotected using 30% piperidine in DMF and the resulting NH_2-dPEG$_4$-AMB was purified by RP-HPLC and its mass was confirmed by mass spectrometry. This NH_2-dPEG$_4$-AMB (1 eq., 50 μmol) was then reacted with Fmoc-TXA (3.3 eq., 164 μmol) activated with HBTU (2.8 eq., 142 μmol) and DIEA (1 eq., 54 μmol) overnight. The sample was then deprotected with 30% piperidine and the product was selectively solubilized using water. **RP-HPLC:** The product TXA-dPEG$_4$-AMB was purified using a semi-preparative Thermo Hypersil GOLD C18 column (5μm, 250 x 10 mm) on 5 minutes 20 – 50% Sol B (methanol +0.1% TFA) gradient. The purified product was rotate evaporated, and the yield was 12% (2 mg). **MS QTOF (ESI+, 220V):** Mass found: 536.396 [M+H]+, 558.386 [M+Na]+; Calculated 536.347 [M+H]+, 558.329 [M+Na]+

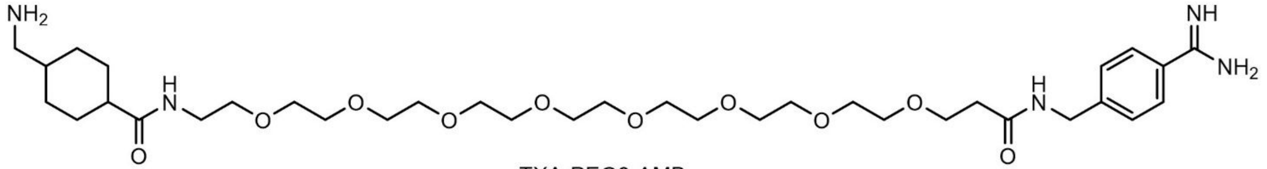

TXA-PEG8-AMB
Chemical Formula: $C_{35}H_{61}N_5O_{10}$
Exact Mass: 711.44

Figure S13: Chemical Structure and characterization of TXA-dPEG$_8$-AMB. To synthesize TXA-dPEG$_8$-AMB, NH_2-dPEG$_8$-AMB was first synthesized by reacting Fmoc-N-amido-dPEG$_8$-NHS (1 eq., 30 µmol) with AMB (3.3 eq., 100 µmol) in a mixture of 70% DMF and 30% 0.01M PBS at pH 7.4. 15uL of TEA was added and the sample was stirred for 30 mins at room temperature. Fmoc was then deprotected using 30% piperidine in DMF and the resulting NH_2-dPEG$_8$-AMB was purified by RP-HPLC and its mass was confirmed by mass spectrometry. This NH_2-dPEG$_8$-AMB (1 eq., 20 µmol) was then reacted with Fmoc-TXA (3 eq., 60 µmol) activated with HBTU (2.6 eq., 52 µmol) and DIEA (1 eq., 20 µmol) overnight. The sample was then deprotected with 30% piperidine and the product was selectively solubilized using water. **RP-HPLC:** The product TXA-dPEG$_8$-AMB was purified using a semi-preparative Thermo Hypersil GOLD C18 column (5µm, 250 x 10 mm) on 5 minutes 20 – 40% Sol B (methanol +0.1% TFA) gradient. The purified product was rotate evaporated, and the yield was 17% (3 mg). **MS QTOF (ESI+, 220V):** Mass found: 712.448 [M+H]+, 734.430 [M+Na]+; Calculated 712.447 [M+H]+, 734.429 [M+Na]+

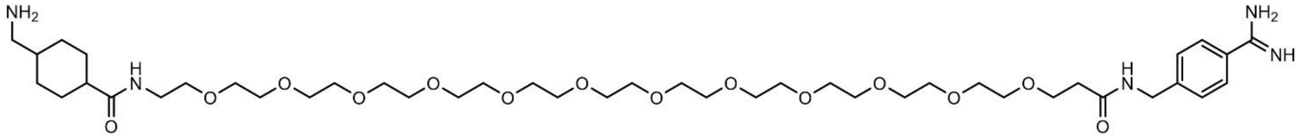

TXA-PEG12-AMB
Chemical Formula: $C_{43}H_{77}N_5O_{14}$
Exact Mass: 887.55

Figure S14: Chemical Structure and characterization of TXA-dPEG$_{12}$-AMB. To synthesize TXA-dPEG$_{12}$-AMB, NH_2-dPEG$_{12}$-AMB was first synthesized by reacting Fmoc-N-amido-dPEG$_{12}$-NHS (1 eq., 27 µmol) with AMB (3 eq., 80 µmol) in a mixture of 70% DMF and 30% 0.01M PBS at pH 7.4. 15uL of TEA was added and the sample was stirred for 30 mins at room temperature. Fmoc was then deprotected using 30% piperidine in DMF and the resulting NH_2-dPEG$_{12}$-AMB was purified by RP-HPLC and its mass was confirmed by mass spectrometry. This NH_2-dPEG$_{12}$-AMB (1 eq., 27 µmol) was then reacted with Fmoc-TXA (4 eq., 108 µmol) activated with HBTU (3.5 eq., 96 µmol) and DIEA (1 eq., 20 µmol) overnight. The sample was then deprotected with 30% piperidine and the product was selectively solubilized using water. **RP-HPLC:** The product TXA-dPEG$_{12}$-AMB was purified using a semi-preparative Thermo Hypersil GOLD C18 column (5µm, 250 x 10 mm) on 5 minutes 30 – 60% Sol B (methanol +0.1% TFA) gradient. The purified product was rotate evaporated, and the yield was 15% (2.9 mg). **MS QTOF (ESI+, 220V):** 888.586 [M+H]+, 910.563 [M+Na]+; Calculated 888.557 [M+H]+, 910.539[M+Na]+

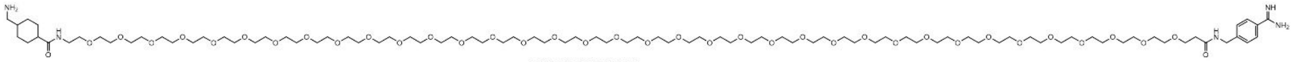

TXA-PEG36-AMB
Chemical Formula: $C_{91}H_{173}N_5O_{38}$
Exact Mass: 1944.18

Figure S15: Chemical Structure and characterization of TXA-dPEG$_{36}$-AMB. To synthesize TXA-dPEG$_{36}$-AMB, NH_2-dPEG$_{36}$-AMB was first synthesized by reacting Fmoc-N-amido-dPEG36-NHS (1 eq., 10 µmol) with AMB (4 eq., 40 µmol) in a mixture of 70% DMF and 30% 0.01M PBS at pH 7.4. 15uL of TEA was added and the sample was stirred for 30 mins at room temperature. Fmoc was then deprotected using 30% piperidine in DMF and the resulting NH_2-dPEG$_{36}$-AMB was purified by RP-HPLC and its mass was confirmed by mass spectrometry. This NH_2-dPEG$_{36}$-AMB (1 eq., 15 µmol) was then reacted with Fmoc-TXA (4 eq., 60 µmol) activated with HBTU (3.5 eq., 53 µmol) and DIEA (1 eq., 20 µmol) overnight. The sample was then deprotected with 30% piperidine and the product was selectively solubilized using water. **RP-HPLC:** The product TXA-dPEG$_{36}$-AMB was purified using a semi-preparative Thermo Hypersil GOLD C18 column (5µm, 250 x 10 mm) on 5 minutes 50 – 70% Sol B (methanol +0.1% TFA) gradient. The purified product was rotate evaporated, and the yield was 8% (2.2 mg). **MS QTOF (ESI+, 220V):** 1945.053 [M+H]+, 1967.032 [M+Na]+; Calculated 1945.187 [M+H]+, 1967.169 [M+Na]+

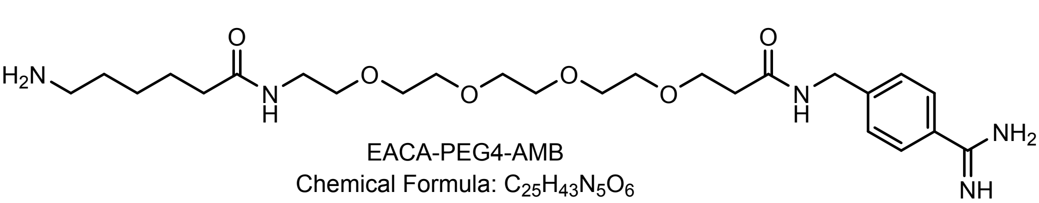

EACA-PEG4-AMB
Chemical Formula: $C_{25}H_{43}N_5O_6$
Exact Mass: 509.32

Figure S16: Chemical Structure and characterization of EACA-dPEG$_4$-AMB. To synthesize EACA-dPEG$_4$-AMB, NH_2-dPEG$_4$-AMB was first synthesized by reacting Fmoc-N-amido-dPEG$_4$-NHS (1 eq., 40 µmol) with AMB (3.25 eq., 130 µmol) in a mixture of 70% DMF and 30% 0.01M PBS at pH 7.4. 15uL of TEA was added and the sample was stirred for 30 mins at room temperature. Fmoc was then deprotected using 30% piperidine in DMF and the resulting NH_2-dPEG$_4$-AMB was purified by RP-HPLC and its mass was confirmed by mass spectrometry. This NH_2-dPEG$_4$-AMB (1 eq., 50 µmol) was then reacted with Fmoc-EACA (3.3 eq., 164 µmol) activated with HBTU (2.8 eq., 142 µmol) and DIEA (1 eq., 54 µmol) overnight. The sample was then deprotected with 30% piperidine and the product was selectively solubilized using water. **RP-HPLC:** The product EACA-dPEG$_4$-AMB was purified using a semi-preparative Thermo Hypersil GOLD C18 column (5µm, 250 x 10 mm) on 5 minutes 20 – 50% Sol B (methanol +0.1% TFA) gradient. The purified product was rotate evaporated, and the yield was 20% (1.3 mg). **MS QTOF (ESI+, 220V):** 510.329 [M+H]+, 532.317 [M+Na]+; Calculated 510.327 [M+H]+, 532.309 [M+Na]+

S4 Inhibition Assays for Hetero-bivalent Inhibitors

S4.1 Plasmin Inhibition with Hetero-bivalent Inhibitors

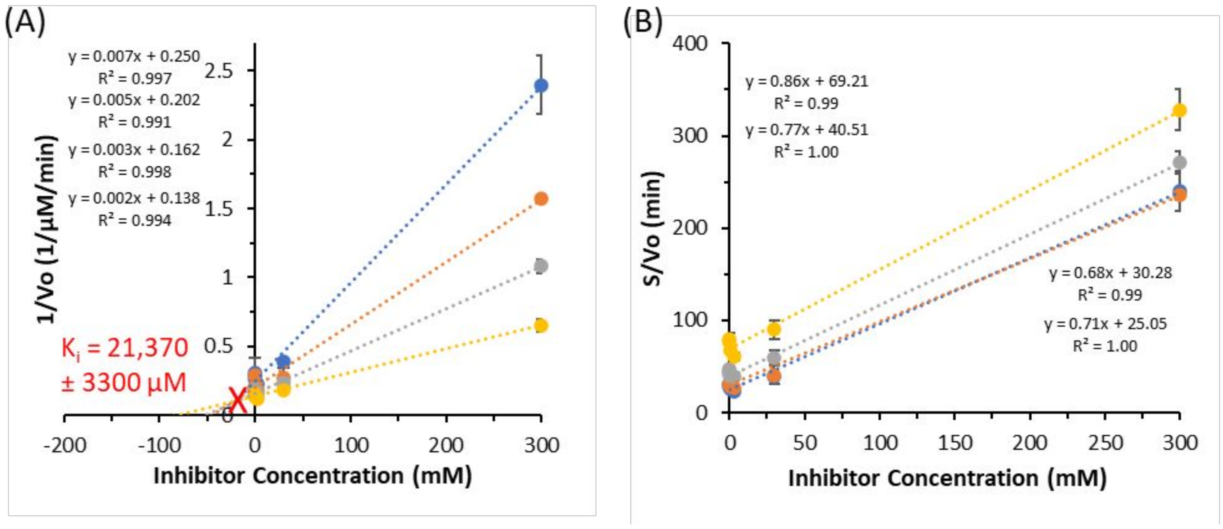

Figure S17: (A) K_i determination of TXA utilizing a Dixon Plot. 0 – 300,000 µM of TXA was incubated with a fixed plasmin concentration of 42.5 nM in PBS pH 7.4. Four different S-2251 concentrations of 100 µM (blue), 150 µM (orange), 250 µM (gray), and 500 µM (yellow) were utilized to obtain K_i which is the negative intersection of the lines at 21,370 ± 3,300 µM. (B) Cornish-Bowden S/Vo vs I plot was used to determine the mode of inhibition. TXA was found to be a weak competitive inhibitor as the lines in this plot are parallel. All data is represented as mean ± SD of triplicate experiments.

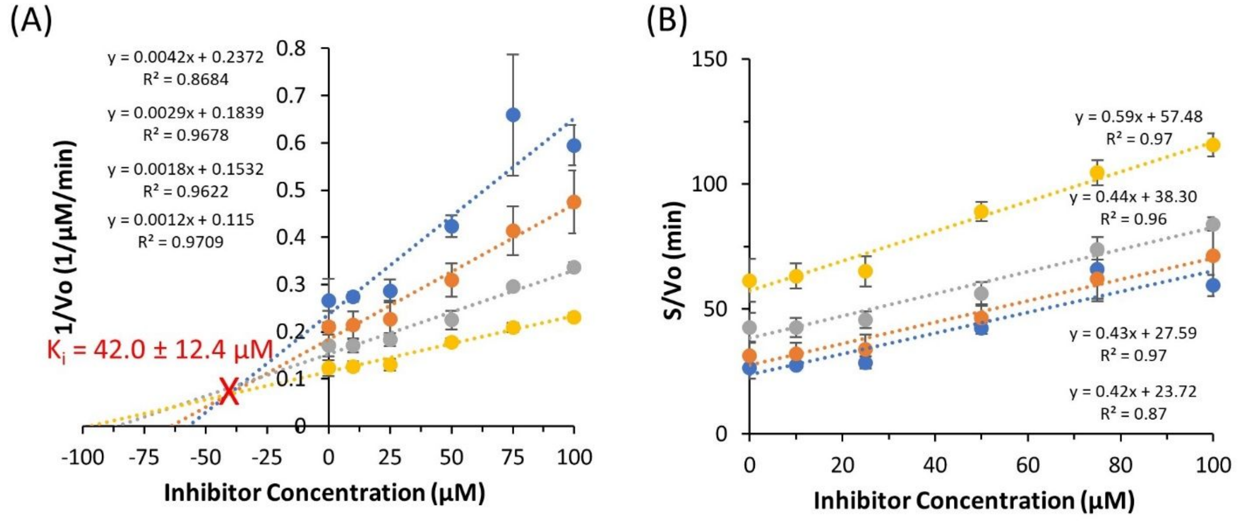

Figure S18: (A) K_i determination of TXA-dPEG$_4$-AMB utilizing a Dixon Plot. 0 – 100 µM of TXA-dPEG$_4$-AMB was incubated with a fixed plasmin concentration of 42.5 nM in PBS pH 7.4. Four different S-2251 concentrations of 100 µM (blue), 150 µM (orange), 250 µM (gray), and 500 µM (yellow) were utilized to obtain K_i which is the negative intersection of the lines at 42.0 ± 12.4 µM. (B) Cornish-Bowden S/Vo vs I plot was used to determine the mode of inhibition. TXA-dPEG$_4$-AMB was found to be a competitive inhibitor as the lines in this plot are parallel. All data is represented as mean ± SD of triplicate experiments.

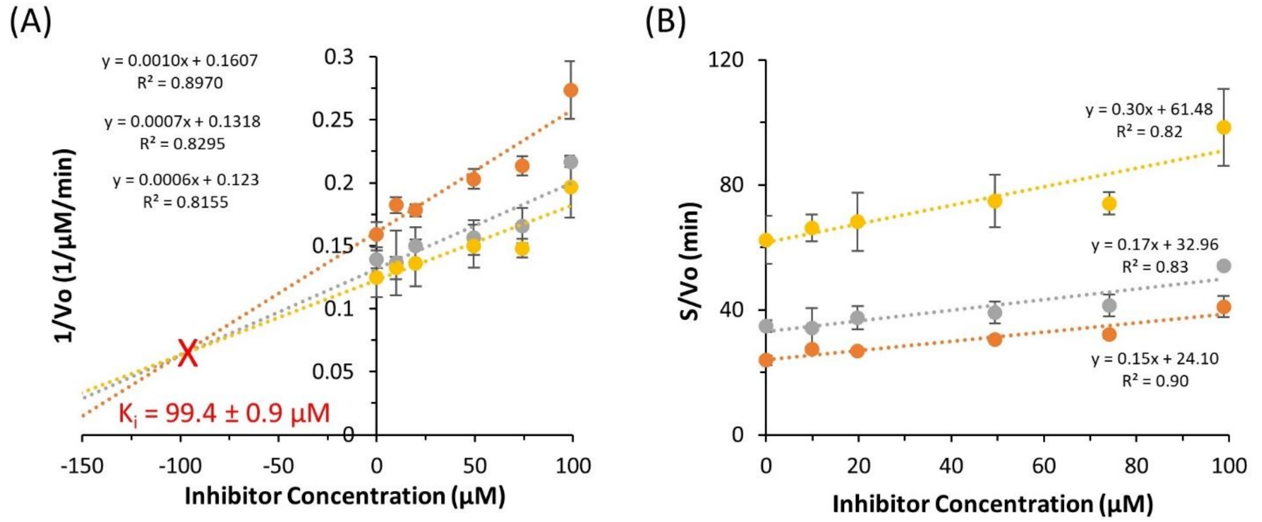

Figure S19: (A) K_i determination of TXA-dPEG$_8$-AMB utilizing a Dixon Plot. 0 – 100 μM of TXA-dPEG$_8$-AMB was incubated with a fixed plasmin concentration of 42.5 nM in PBS pH 7.4. Three different S-2251 concentrations of 150 μM (orange), 250 μM (gray), and 500 μM (yellow) were utilized to obtain K_i which is the negative intersection of the lines at 99.4 ± 0.9 μM. (B) Cornish-Bowden S/Vo vs I plot was used to determine the mode of inhibition. TXA-dPEG$_8$-AMB was found to be a competitive inhibitor as the lines in this plot are parallel. All data is represented as mean ± SD of triplicate experiments.

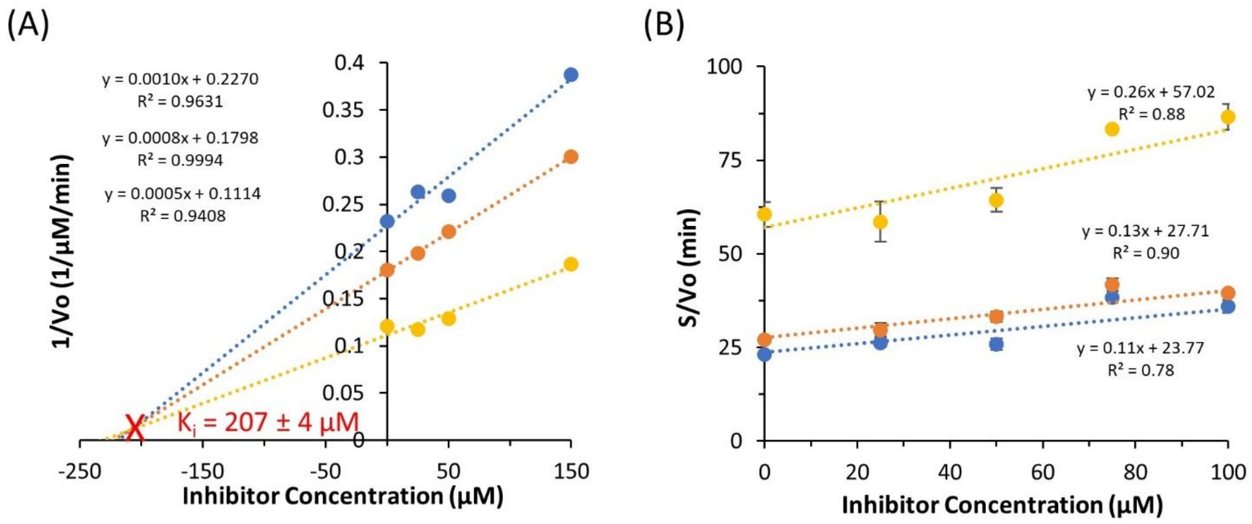

Figure S20: (A) K_i determination of TXA-dPEG$_{12}$-AMB utilizing a Dixon Plot. 0 – 150 μM of TXA-dPEG$_{12}$-AMB was incubated with a fixed plasmin concentration of 42.5 nM in PBS pH 7.4. Three different S-2251 concentrations of 100 μM (blue), 150 μM (orange) and 500 μM (yellow) were utilized to obtain K_i which is the negative intersection of the lines at 207 ± 4 μM. (B) Cornish-Bowden S/Vo vs I plot was used to determine the mode of inhibition. TXA-dPEG$_{12}$-AMB was found to be a competitive inhibitor as the lines in this plot are parallel. All data is represented as mean ± SD of triplicate experiments.

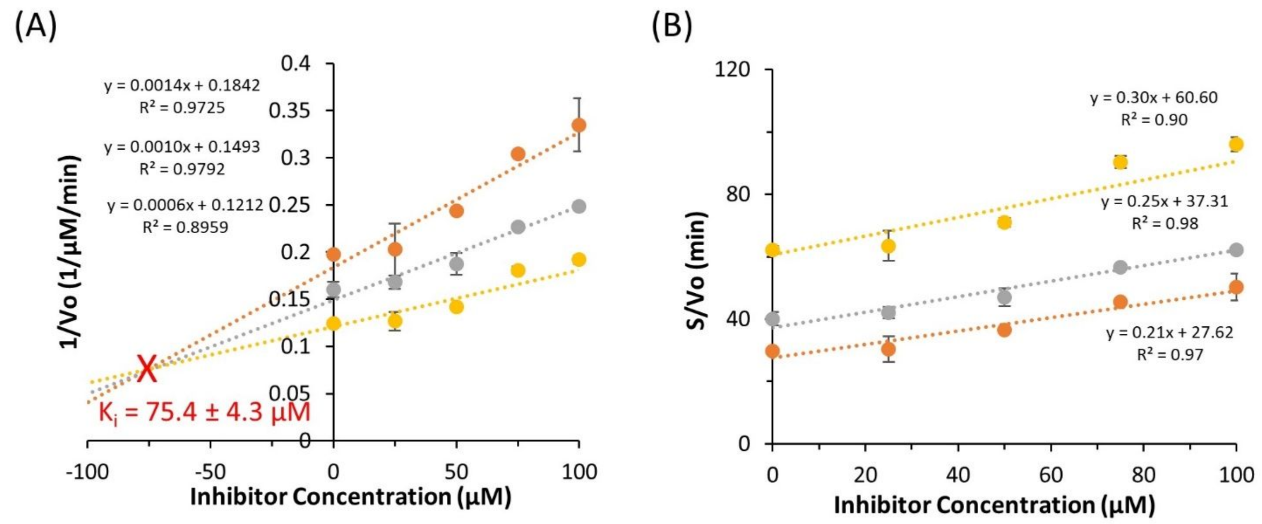

Figure S21: (A) K_i determination of TXA-dPEG$_{36}$-AMB utilizing a Dixon Plot. 0 – 100 µM of TXA-dPEG$_{36}$-AMB was incubated with a fixed plasmin concentration of 42.5 nM in PBS pH 7.4. Three different S-2251 concentrations of 150 µM (orange), 300 µM (gray), and 500 µM (yellow) were utilized to obtain K_i which is the negative intersection of the lines at 75.4 ± 4.3 µM. (B) Cornish-Bowden S/Vo vs I plot was used to determine the mode of inhibition. TXA-dPEG$_{36}$-AMB was found to be a competitive inhibitor as the lines in this plot are parallel. All data is represented as mean ± SD of triplicate experiments.

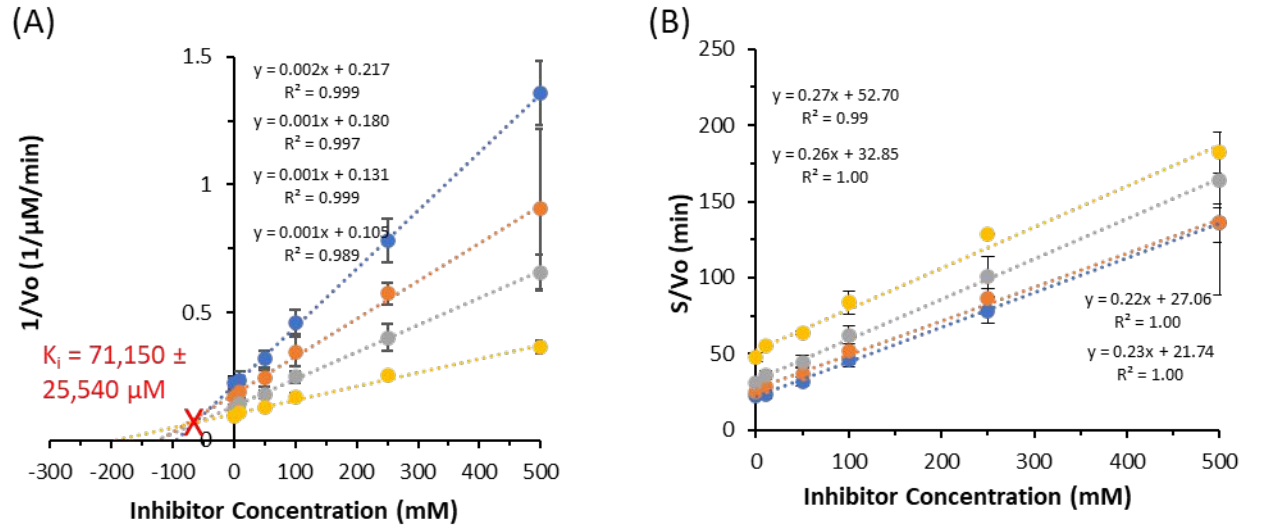

Figure S22: (A) K_i determination of EACA utilizing a Dixon Plot. 0 – 500,000 µM of EACA was incubated with a fixed plasmin concentration of 42.5 nM in PBS pH 7.4. Four different S-2251 concentrations of 100 µM (blue), 150 µM (orange), 250 µM (gray), and 500 µM (yellow) were utilized to obtain K_i which is the negative intersection of the lines at 71,150 ± 25,540 µM. (B) Cornish-Bowden S/Vo vs I plot was used to determine the mode of inhibition. EACA was found to be a weak competitive inhibitor as the lines in this plot are parallel. All data is represented as mean ± SD of triplicate experiments.

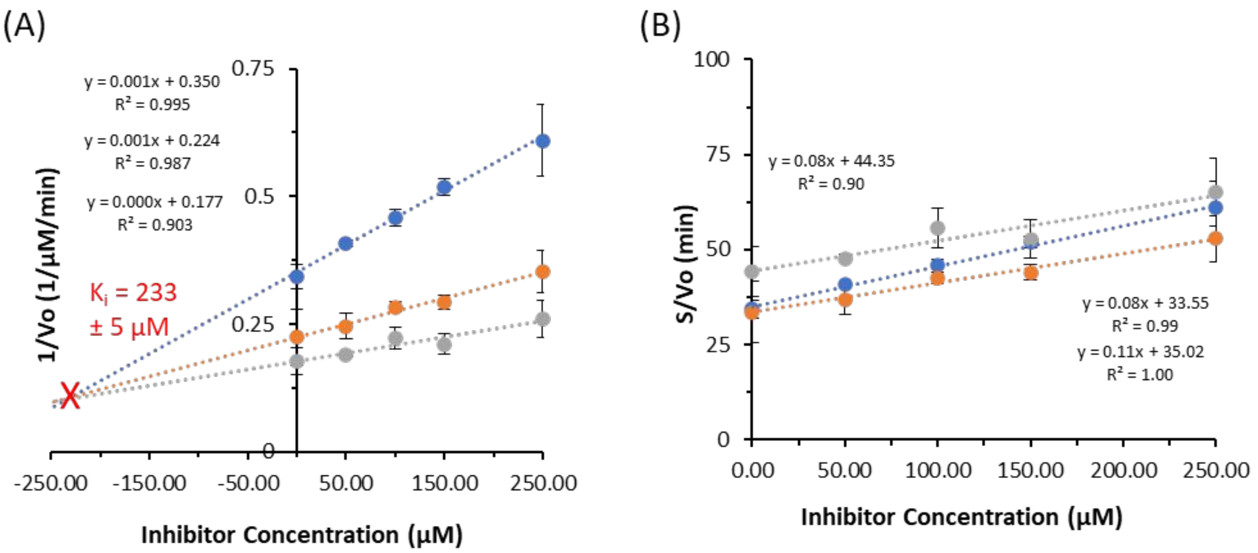

Figure S23: (A) K_i determination of EACA-dPEG$_4$-AMB utilizing a Dixon Plot. 0 – 250 µM of EACA-dPEG$_4$-AMB was incubated with a fixed plasmin concentration of 42.5 nM in PBS pH 7.4. Three different S-2251 concentrations of 100 µM (blue), 150 µM (orange) and 250 µM (gray) were utilized to obtain K_i which is the negative intersection of the lines at 233 ± 5 µM. (B) Cornish-Bowden S/Vo vs I plot was used to determine the mode of inhibition. EACA-dPEG$_4$-AMB was found to be a competitive inhibitor as the lines in this plot are parallel. All data is represented as mean ± SD of triplicate experiments.

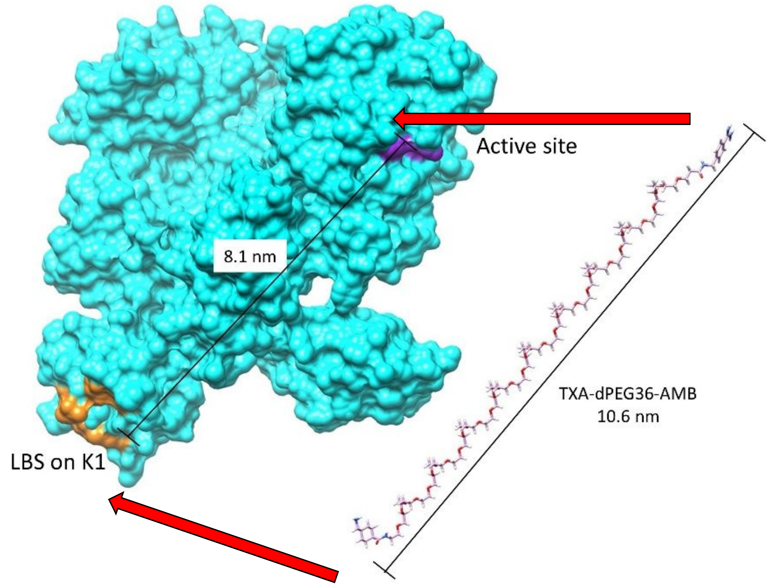

Figure S24: Chimera figure of plasmin and TXA-dPEG$_{36}$-AMB. Theoretical linear separation distance between the active site and LBS on K1 in full-length type II Human plasminogen (PDB ID: 4DUR) was measured to be 8.1 nm (UCSF Chimera, version 1.14). The length of TXA-dPEG$_{36}$-AMB was found to be 10.6 nm, indicating it is longer than the separation distance and can potentially achieve subsite binding by simultaneous binding of benzamidine to the active site and TXA to LBS on K1.

S4.2 tPA inhibition with hetero-bivalent inhibitors

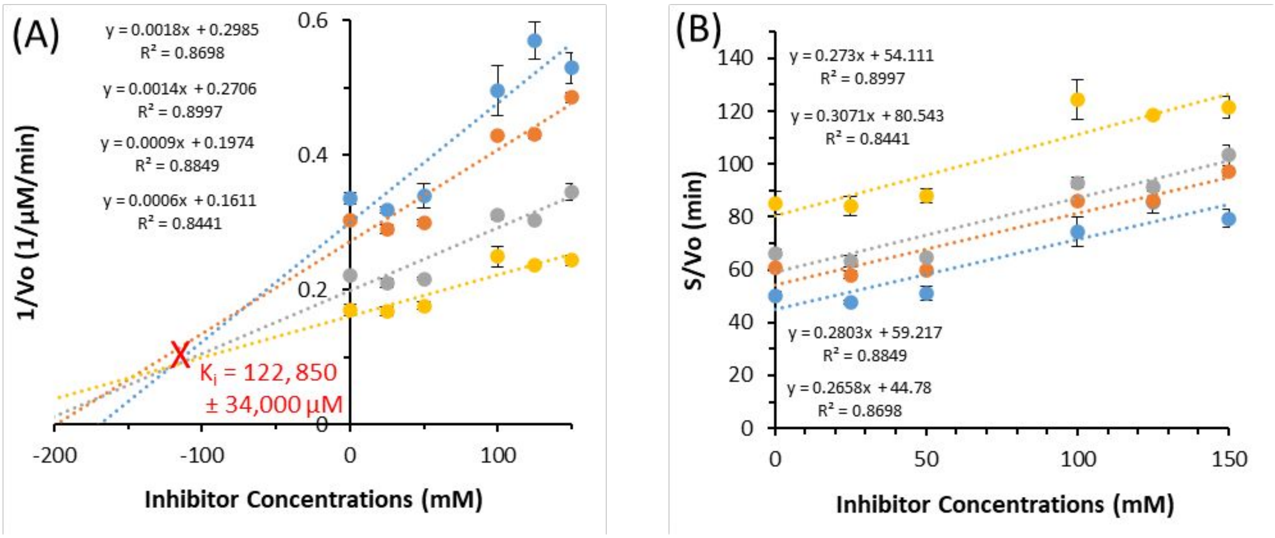

Figure S25: (A) K_i determination of TXA utilizing a Dixon Plot. 0 – 150,000 µM of TXA was incubated with a fixed tPA concentration of 75 nM in PBS pH 7.4. Four different S-2288 concentrations of 150 µM (blue), 200 µM (orange), 300 µM (gray), and 500 µM (yellow) were utilized to obtain K_i which is the negative intersection of the lines at 122,850 ± 34,000 µM. (B) Cornish-Bowden S/Vo vs I plot was used to determine the mode of inhibition. TXA was found to be a weak competitive inhibitor as the lines in this plot are parallel. All data is represented as mean ± SD of triplicate experiments.

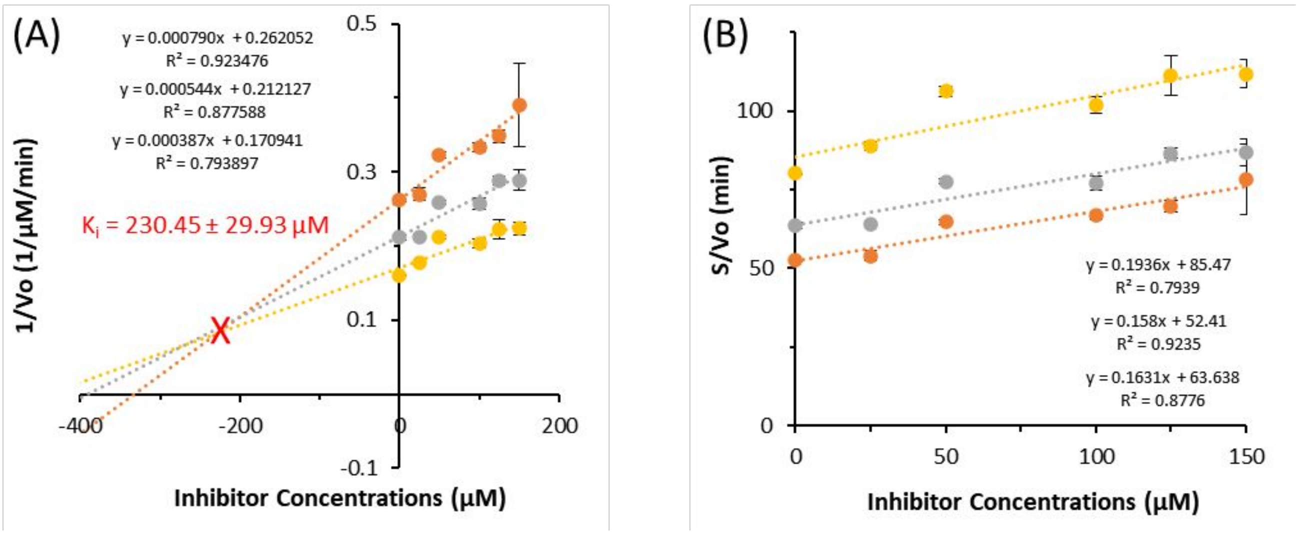

Figure S26: (A) K_i determination of TXA-dPEG$_4$-AMB utilizing a Dixon Plot. 0 – 150 µM of TXA-dPEG$_4$-AMB was incubated with a fixed tPA concentration of 75 nM in PBS pH 7.4. Three different S-2258 concentrations of 200 µM (orange), 300 µM (gray), and 500 µM (yellow) were utilized to obtain K_i which is the negative intersection of the lines at 230.45 ± 29.93 µM. (B) Cornish-Bowden S/Vo vs I plot was used to determine the mode of inhibition. TXA-dPEG$_4$-AMB was found to be a competitive inhibitor as the lines in this plot are parallel. All data is represented as mean ± SD of triplicate experiments.

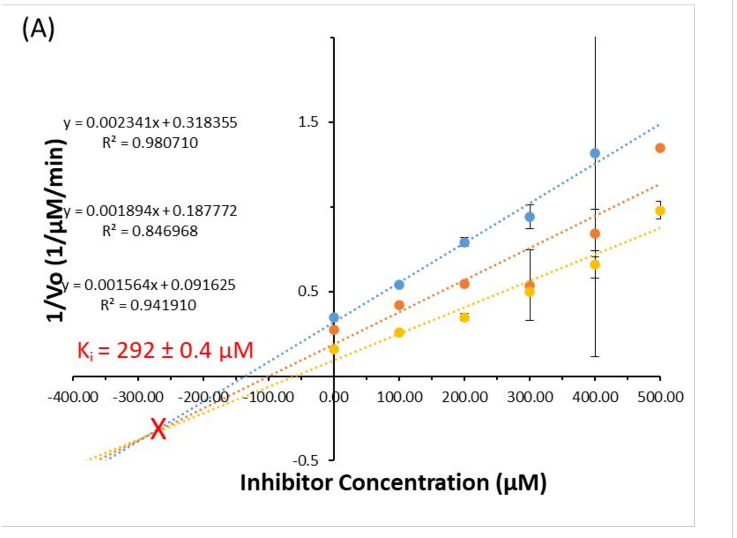

Figure S27: (A) K_i determination of TXA-dPEG$_8$-AMB utilizing a Dixon Plot. 0 – 500 µM of TXA-dPEG$_8$-AMB was incubated with a fixed tPA concentration of 75 nM in PBS pH 7.4. Three different S-2258 concentrations of 100 µM (blue), 200 µM (orange), and 500 µM (yellow) were utilized to obtain K_i which is the negative intersection of the lines at 292 ± 0.4 µM. (B) Cornish-Bowden S/Vo vs I plot was used to determine the mode of inhibition. TXA-dPEG$_8$-AMB was found not to be a solely competitive inhibitor with potential allosteric contributions as the lines in this plot are not parallel. All data is represented as mean ± SD of triplicate experiments.

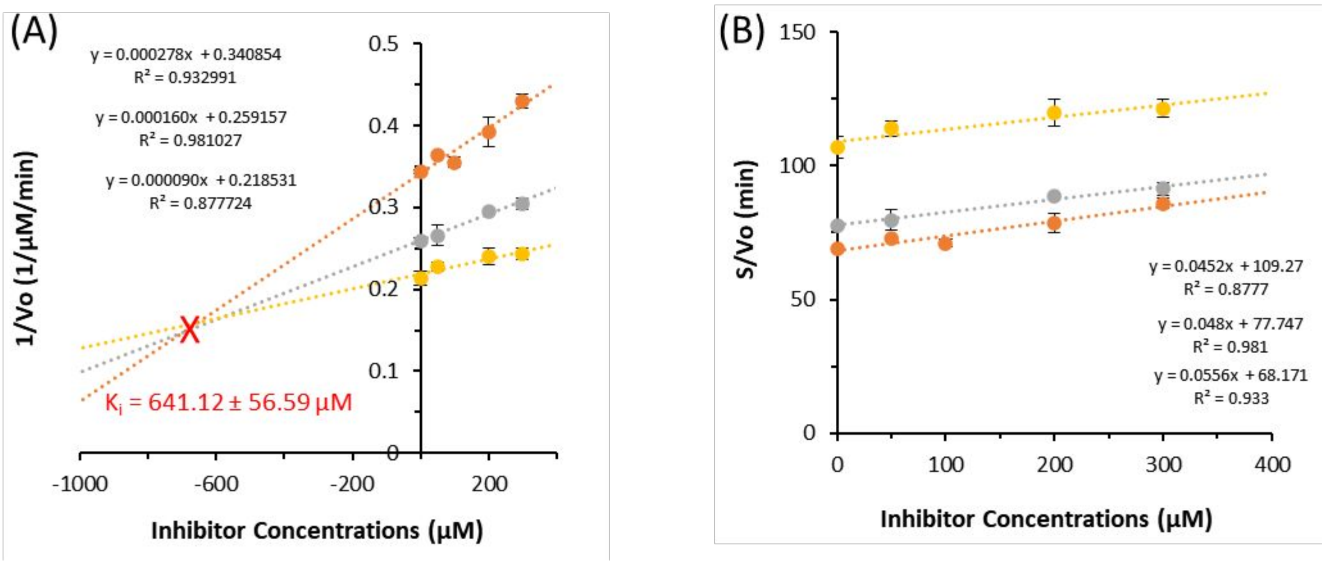

Figure S28: (A) K_i determination of TXA-dPEG$_{12}$-AMB utilizing a Dixon Plot. 0 – 300 µM of TXA-dPEG$_{12}$-AMB was incubated with a fixed tPA concentration of 75 nM in PBS pH 7.4. Three different S-2258 concentrations of 200 µM (orange), 300 µM (gray), and 500 µM (yellow) were utilized to obtain K_i which is the negative intersection of the lines at 641.12 ± 56.59 µM. (B) Cornish-Bowden S/Vo vs I plot was used to determine the mode of inhibition. TXA-dPEG$_{12}$-AMB was found to be a competitive inhibitor as the lines in this plot are parallel. All data is represented as mean ± SD of triplicate experiments.

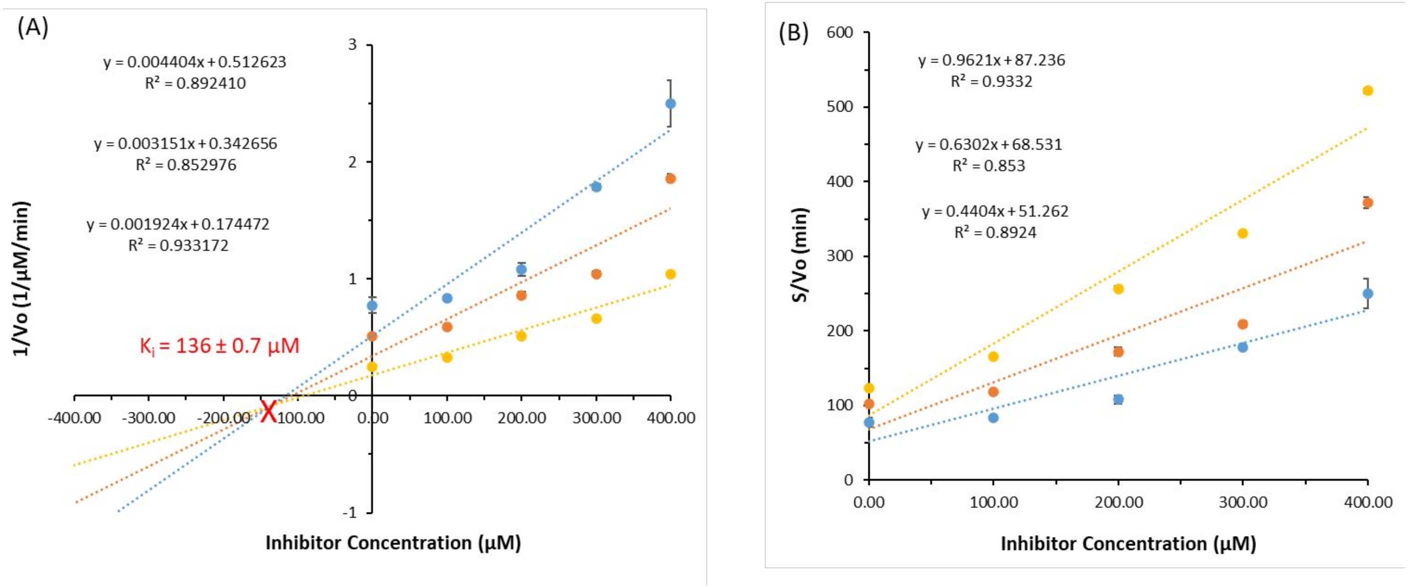

Figure S29: (A) K_i determination of TXA-dPEG$_{36}$-AMB utilizing a Dixon Plot. 0 – 400 μM of TXA-dPEG$_{36}$-AMB was incubated with a fixed tPA concentration of 75 nM in PBS pH 7.4. Three different S-2258 concentrations of 100 μM (blue), 200 μM (orange), and 500 μM (yellow) were utilized to obtain K_i which is the negative intersection of the lines at 136 ± 0.7 μM. (B) Cornish-Bowden S/Vo vs I plot was used to determine the mode of inhibition. TXA-dPEG$_{36}$-AMB was found not to be a solely competitive inhibitor with potential allosteric contributions as the lines in this plot are not parallel. All data is represented as mean ± SD of triplicate experiments.

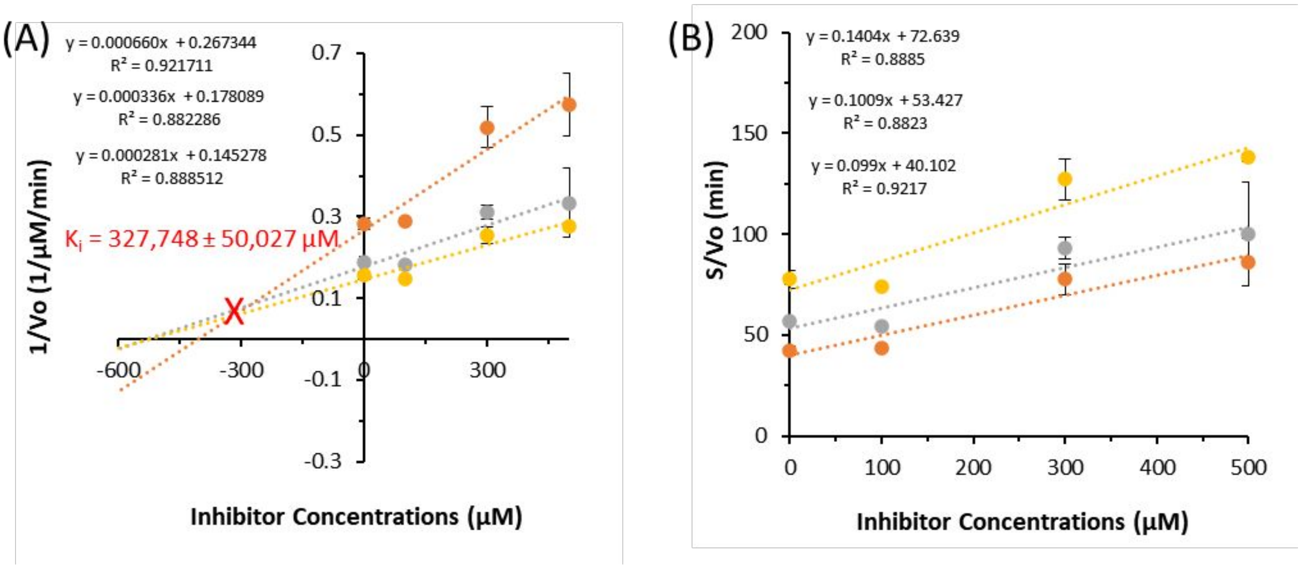

Figure S30: (A) K_i determination of EACA utilizing a Dixon Plot. 0 – 500,000 μM of EACA was incubated with a fixed tPA concentration of 75 nM in PBS pH 7.4. Four different S-2258 concentrations of 150 μM (orange), 300 μM (gray), and 500 μM (yellow) were utilized to obtain K_i which is the negative intersection of the lines at 327,748 ± 50,027 μM. (B) Cornish-Bowden S/Vo vs I plot was used to determine the mode of inhibition. EACA was found to be a weak competitive inhibitor as the lines in this plot are parallel. All data is represented as mean ± SD of triplicate experiments.

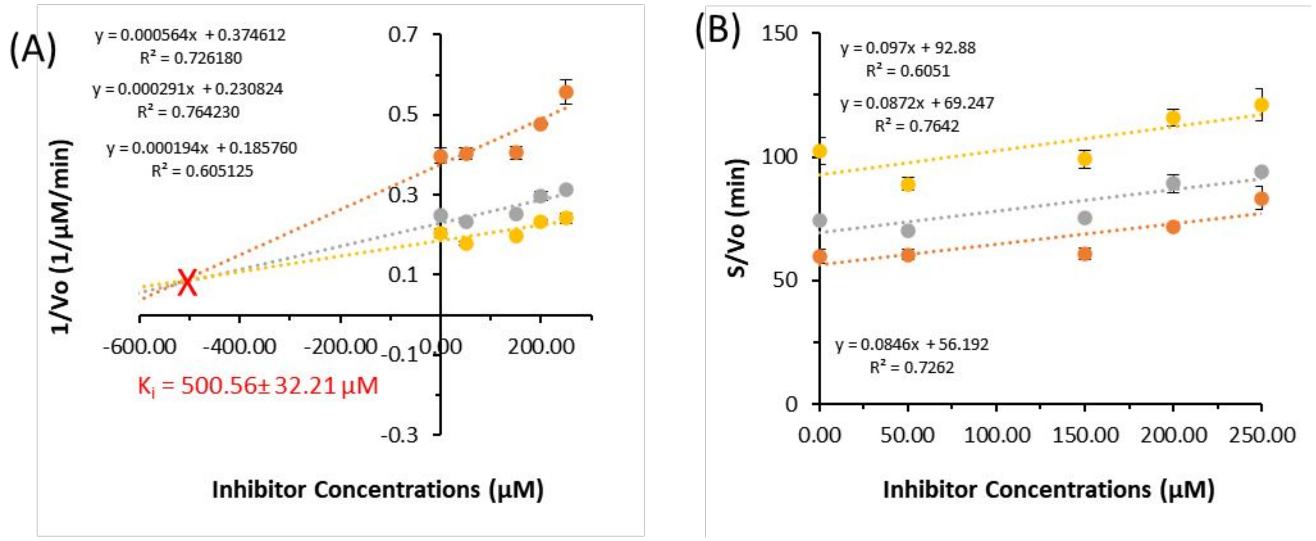

Figure S31: (A) K_i determination of EACA-dPEG$_4$-AMB utilizing a Dixon Plot. 0 – 250 µM of EACA-dPEG$_4$-AMB was incubated with a fixed tPA concentration of 75 nM in PBS pH 7.4. Three different S-2258 concentrations of 150 µM (orange), 300 µM (gray), and 500 µM (yellow) were utilized to obtain K_i which is the negative intersection of the lines at 500.56 ± 32.21 µM. (B) Cornish-Bowden S/Vo vs I plot was used to determine the mode of inhibition. EACA-dPEG$_4$-AMB was found to be a competitive inhibitor as the lines in this plot are parallel. All data is represented as mean ± SD of triplicate experiments.

S4.3 Thrombin inhibition with hetero-bivalent inhibitors

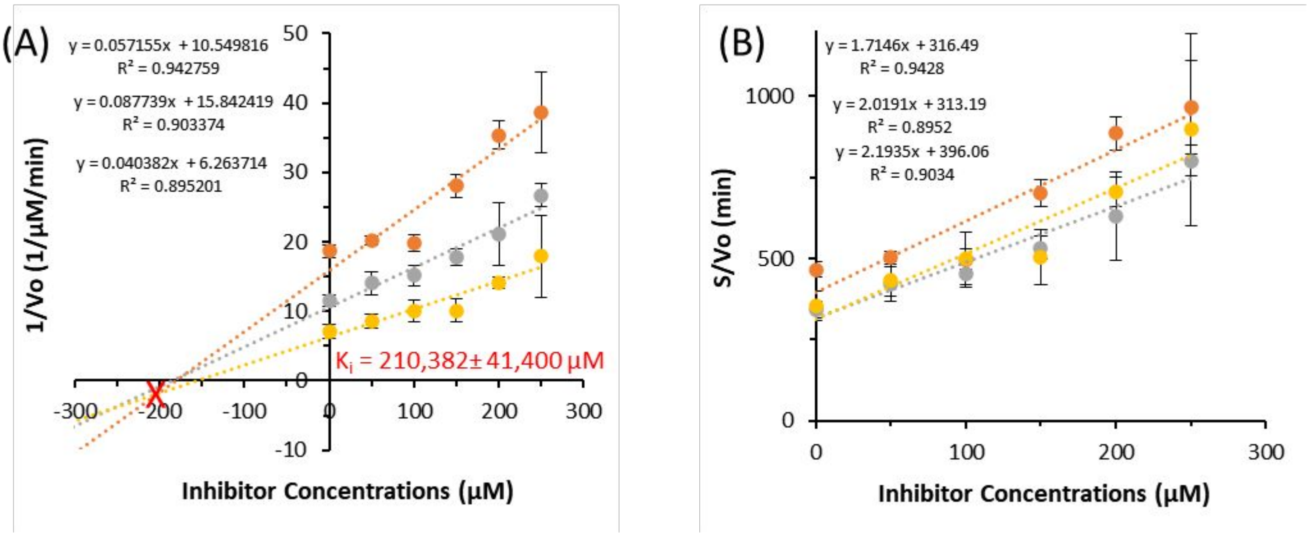

Figure S32: (A) K_i determination of TXA utilizing a Dixon Plot. 0 – 250,000 µM of TXA was incubated with a fixed thrombin concentration of 0.25 u/mL in PBS pH 7.4 at 10% DMSO. Three different TSIII concentrations of 25 µM (orange), 30 µM (gray), and 50 µM (yellow) were utilized to obtain K_i which is the negative intersection of the lines at 210,382 ± 41,400 µM. (B) Cornish-Bowden S/Vo vs I plot was used to determine the mode of inhibition. TXA was found to be a weak competitive inhibitor as the lines in this plot are parallel. All data is represented as mean ± SD of triplicate experiments.

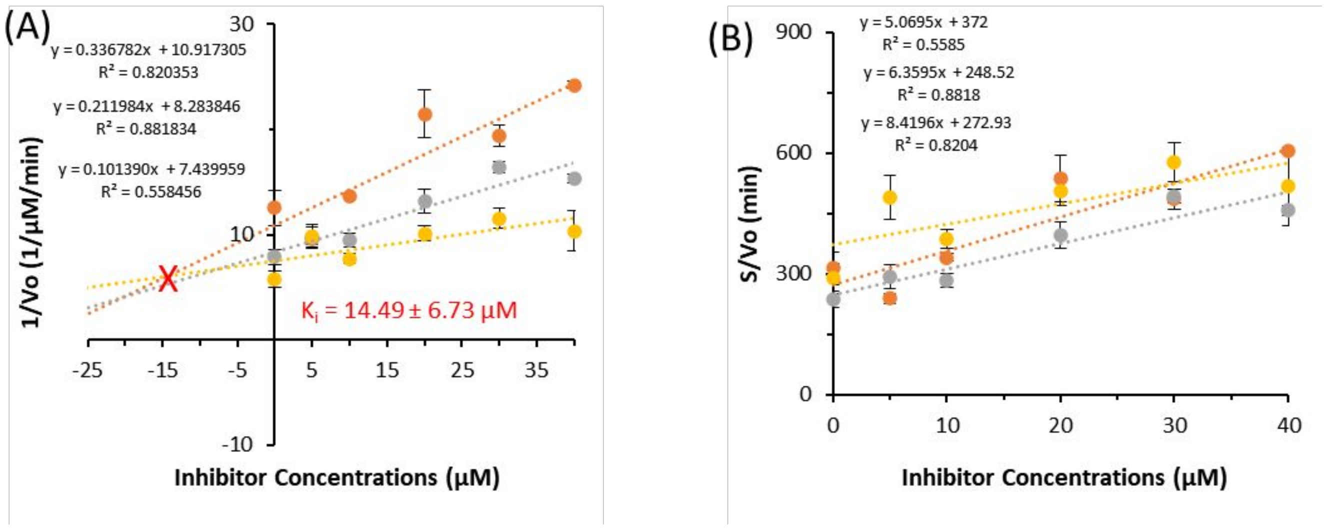

Figure S33: (A) K_i determination of TXA-dPEG$_4$-AMB utilizing a Dixon Plot. 0 – 40 µM of TXA-dPEG$_4$-AMB was incubated with a fixed thrombin concentration of 0.25 u/mL in PBS pH 7.4 at 10% DMSO. Three different TSIII concentrations of 25 µM (orange), 30 µM (gray), and 50 µM (yellow) were utilized to obtain K_i which is the negative intersection of the lines at 14.49 ± 6.73 µM. (B) Cornish-Bowden S/Vo vs I plot was used to determine the mode of inhibition. TXA-dPEG$_4$-AMB was found to be a competitive inhibitor as the lines in this plot are parallel. All data is represented as mean ± SD of triplicate experiments.

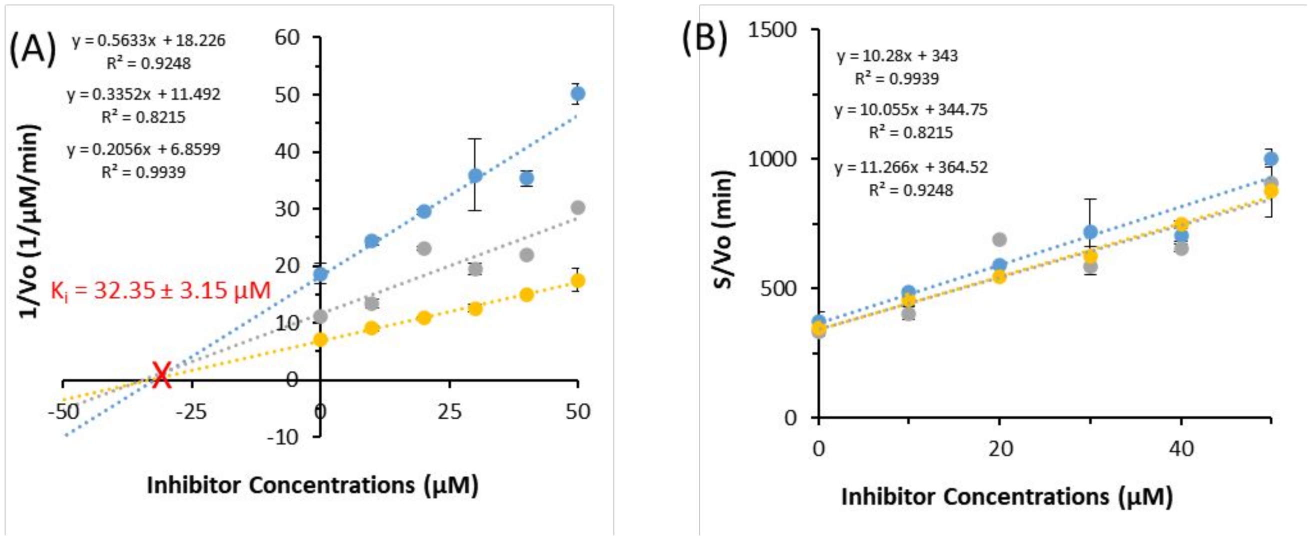

Figure S34: (A) K_i determination of TXA-dPEG$_8$-AMB utilizing a Dixon Plot. 0 – 50 µM of TXA-dPEG$_8$-AMB was incubated with a fixed thrombin concentration of 0.25 u/mL in PBS pH 7.4 at 10% DMSO. Three different TSIII concentrations of 20 µM (blue), 30 µM (gray), and 50 µM (yellow) were utilized to obtain K_i which is the negative intersection of the lines at 32.35 ± 3.15 µM. (B) Cornish-Bowden S/Vo vs I plot was used to determine the mode of inhibition. TXA-dPEG$_8$-AMB was found to be a competitive inhibitor as the lines in this plot are parallel. All data is represented as mean ± SD of triplicate experiments.

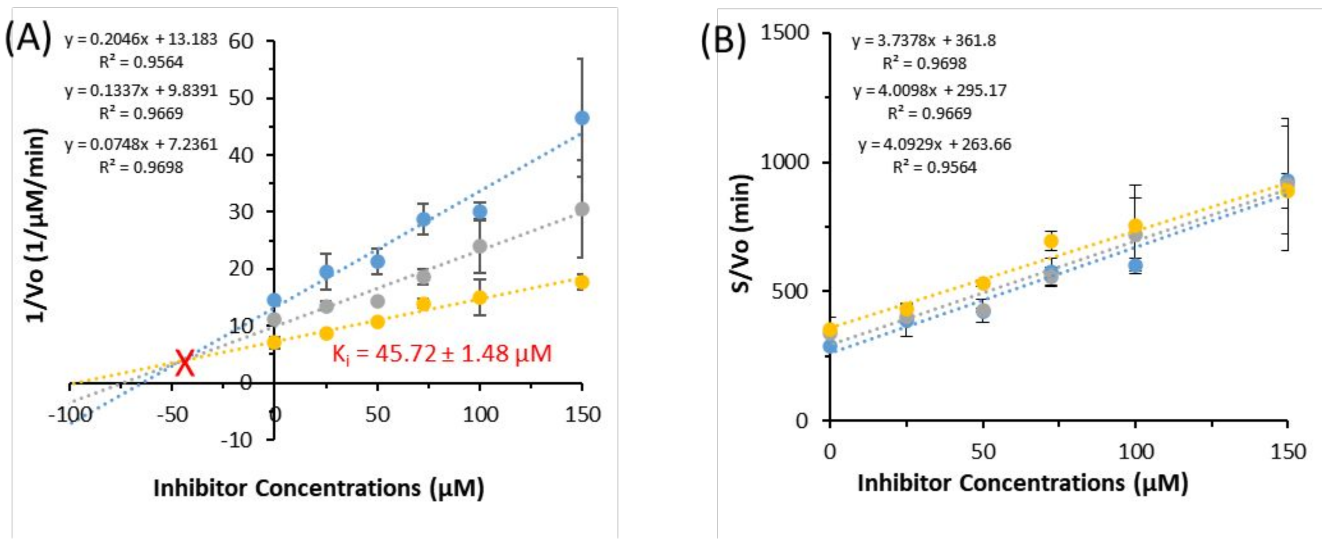

Figure S35: (A) K_i determination of TXA-dPEG$_{12}$-AMB utilizing a Dixon Plot. 0 – 150 µM of TXA-dPEG$_{12}$-AMB was incubated with a fixed thrombin concentration of 0.25 u/mL in PBS pH 7.4 at 10% DMSO. Three different TSIII concentrations of 20 µM (blue), 30 µM (gray), and 50 µM (yellow) were utilized to obtain K_i which is the negative intersection of the lines at 45.72 ± 1.48 µM. (B) Cornish-Bowden S/Vo vs I plot was used to determine the mode of inhibition. TXA-dPEG$_{12}$-AMB was found to be a competitive inhibitor as the lines in this plot are parallel. All data is represented as mean ± SD of triplicate experiments.

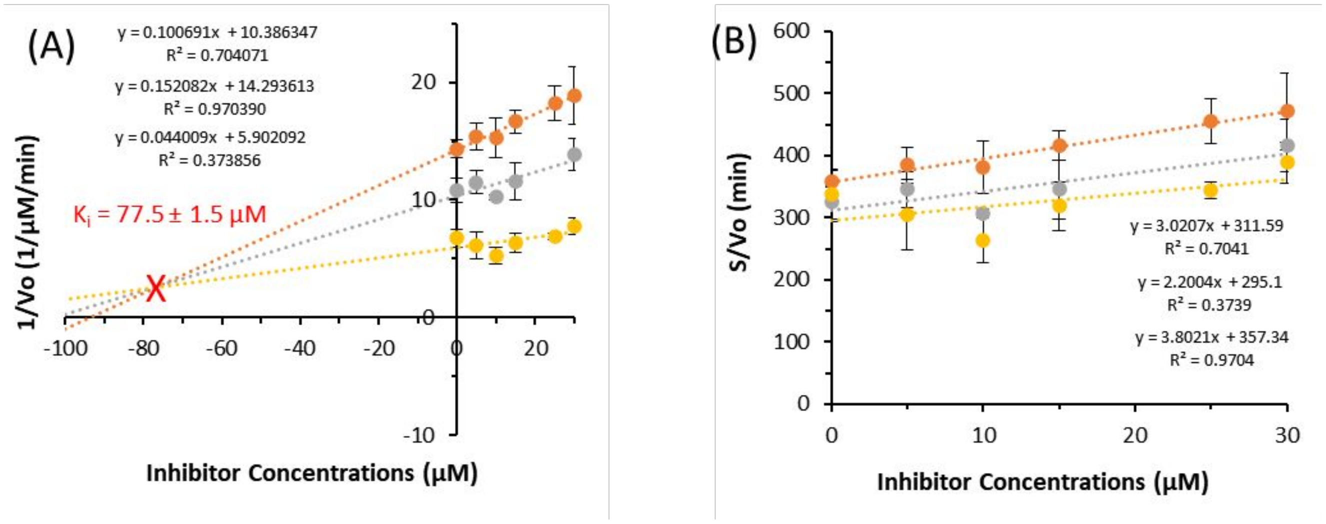

Figure S36: (A) K_i determination of TXA-dPEG$_{36}$-AMB utilizing a Dixon Plot. 0 – 30 µM of TXA-dPEG$_{36}$-AMB was incubated with a fixed thrombin concentration of 0.25 u/mL in PBS pH 7.4 at 10% DMSO. Three different TSIII concentrations of 25 µM (orange), 30 µM (gray), and 50 µM (yellow) were utilized to obtain K_i which is the negative intersection of the lines at 77.5 ± 1.5 µM. (B) Cornish-Bowden S/Vo vs I plot was used to determine the mode of inhibition. TXA-dPEG$_{36}$-AMB was found to be a competitive inhibitor as the lines in this plot are parallel. All data is represented as mean ± SD of triplicate experiments.

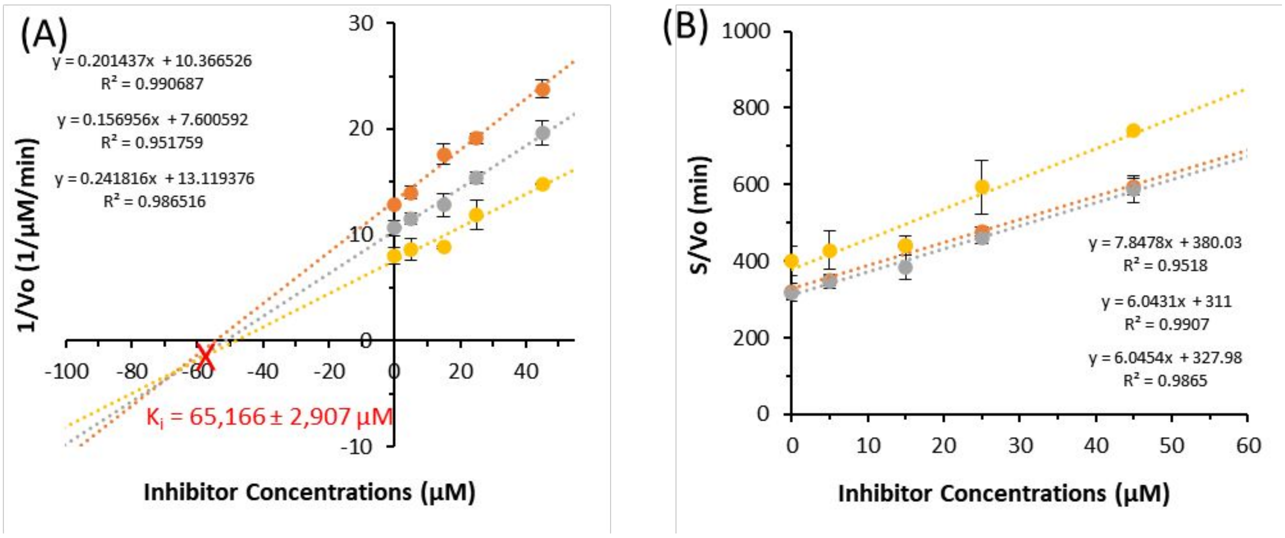

Figure S37: (A) K_i determination of EACA utilizing a Dixon Plot. 0 – 45 µM of EACA was incubated with a fixed thrombin concentration of 0.25 u/mL in PBS pH 7.4 at 10% DMSO. Three different TSIII concentrations of 25 µM (orange), 30 µM (gray), and 50 µM (yellow) were utilized to obtain K_i which is the negative intersection of the lines at 65,166 ± 2,907 µM. (B) Cornish-Bowden S/Vo vs I plot was used to determine the mode of inhibition. EACA was found to be a competitive inhibitor as the lines in this plot are parallel. All data is represented as mean ± SD of triplicate experiments.

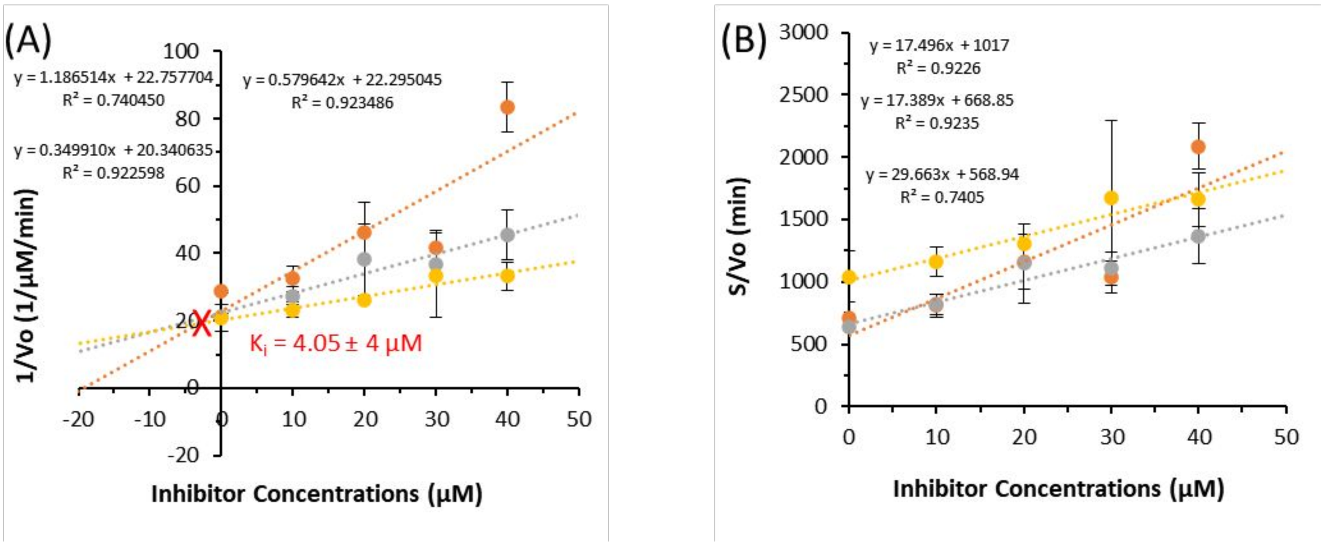

Figure S38: (A) K_i determination of EACA-dPEG$_4$-AMB utilizing a Dixon Plot. 0 – 40 µM of EACA-dPEG$_4$-AMB was incubated with a fixed thrombin concentration of 0.25 u/mL in PBS pH 7.4 at 10% DMSO. Three different TSIII concentrations of 25 µM (orange), 30 µM (gray), and 50 µM (yellow) were utilized to obtain K_i which is the negative intersection of the lines at 4.05 ± 4 µM. (B) Cornish-Bowden S/Vo vs I plot was used to determine the mode of inhibition. EACA-dPEG$_4$-AMB was found to be a competitive inhibitor as the lines in this plot are parallel. All data is represented as mean ± SD of triplicate experiments.

S5 Synthesis and Characterization of Homo-multivalent TXA Inhibitors

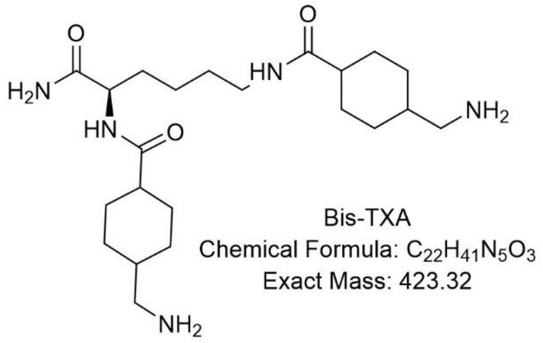

Figure S39: Chemical Structure and characterization of Bis-TXA. Bis-TXA was synthesized on NovaPEG rink amide resin using Solid Phase Peptide Synthesis (SPPS) method. First, Fmoc-Lys(Fmoc)-OH (4 eq., 100 µmol) activated with HBTU (3.6 eq., 90 µmol) and DIEA (8 eq., 200 µmol) was conjugated to the resin in DMF at room temperature for 2 hours. After confirming the conjugation with a Kaiser test, Fmoc was deprotected using 20% piperidine in DMF. To this, Fmoc-TXA (8 eq., 200 µmol) activated with HBTU (7.2 eq., 180 µmol) and DIEA (16 eq., 400 µmol) in DMF was added and the reaction was run overnight at room temperature. After confirming Fmoc-TXA conjugation with a Kaiser test, Fmoc on TXA was deprotected using 20% piperidine in DMF. Finally, the product was cleaved from the resin using TFA cleavage cocktail (95% TFA/ 2.5% TIS/ 2.5% water) and was dried using a rotate evaporator. **RP-HPLC:** Bis-TXA was purified using semi-preparative Thermo Hypersil GOLD C18 column (5µm, 250 x 10 mm) on 5 minutes 10 – 20% Sol B (methanol+0.1% TFA) gradient.The purified product was rotate evaporated, and the yield was 27% (2.8 mg). **MS QTOF (ESI+, 220V):** Mass found: 424.4718 $[M+H]^+$, 446.4694 $[M+Na]^+$; Calculated 424.3273 $[M+H]^+$, 446.3092 $[M+Na]^+$

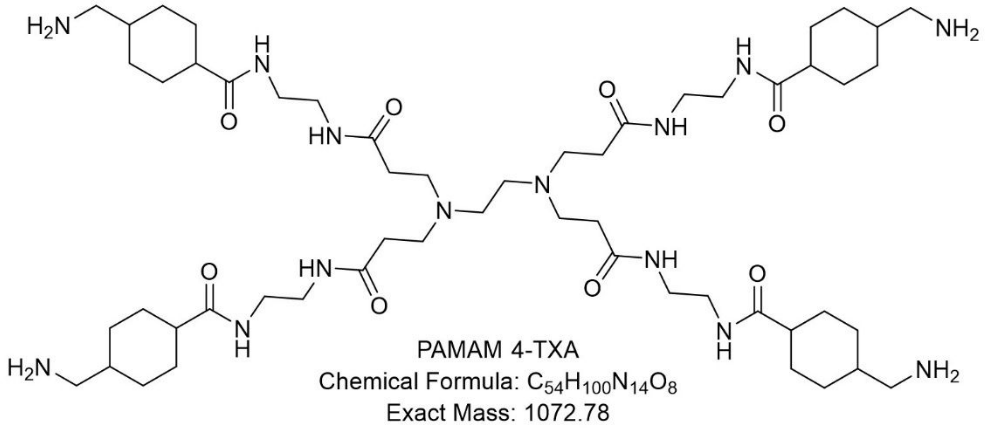

Figure S40: Chemical Structure and characterization of PAMAM^4-TXA. PAMAM^4-TXA was synthesized by reacting PAMAM^4 Dendrimer, Generation 0 (1 eq., 39 µmol) with Fmoc-TXA (12 eq., 465 µmol) using HBTU (10.8 eq., 418 µmol), Oxyma Pure (10.8 eq., 418 µmol) and DIEA (24 eq., 930 µmol) in DMF for 4 hours while stirring at room temperature. The sample was then precipitated out using cold diethylether and Fmoc was deprotected using 30% piperidine in DMF. The sample was again precipitated out using cold diethylether. The precipitate was then solubilized. **RP-HPLC:** PAMAM^4-TXA was then purified using semi-preparative Thermo Hypersil GOLD C18 column (5µm, 250 x 10 mm) on 5 minutes 10 – 25% Sol B (methanol+0.1% TFA) gradient. The purified product was rotate evaporated, and the yield was 20% (8.2 mg). **MS QTOF (ESI+, 220V):** Mass found: 1073.8285 $[M+H]^+$, 537.4168 $[M+2H]^{2+}$; Calculated 1073.7873 $[M+H]^+$, 537.3973 $[M+2H]^{2+}$

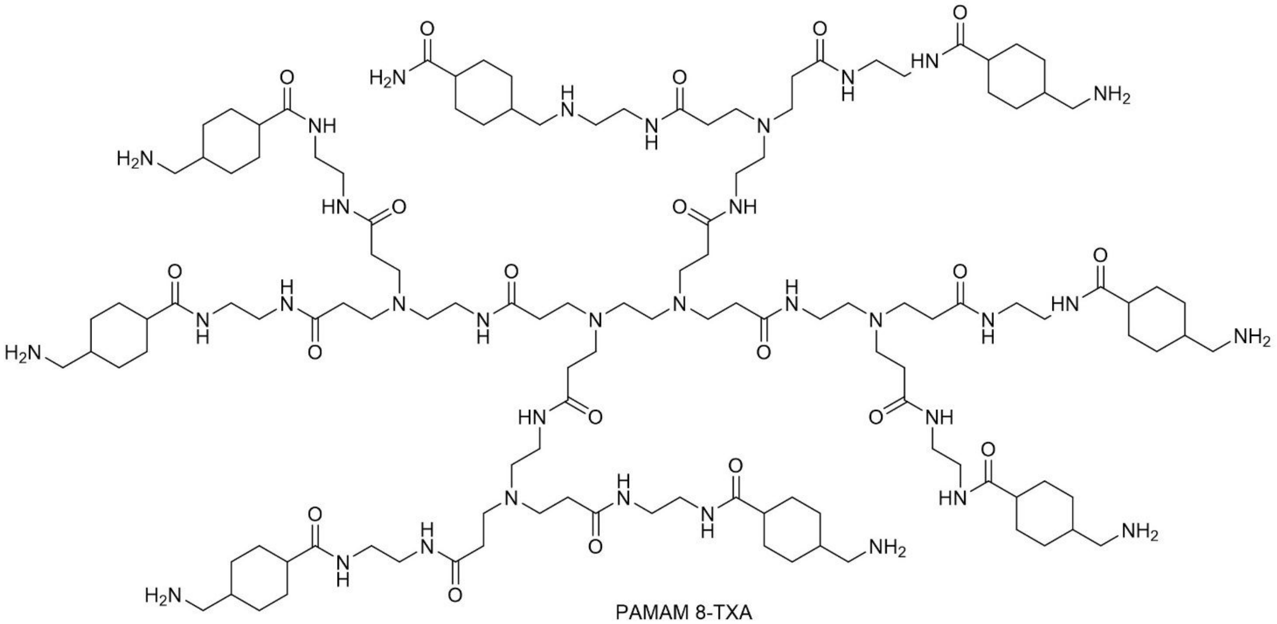

Figure S41: Chemical Structure and characterization of PAMAM[8]-TXA. PAMAM[8]-TXA was synthesized by reacting PAMAM[8] Dendrimer, Generation 1 (1 eq., 17 µmol) with Fmoc-TXA (18 eq., 315 µmol) using HBTU (16.2 eq., 283 µmol), Oxyma Pure (18 eq., 283 µmol) and DIEA (40 eq., 680 µmol) in DMF for 4 hours while stirring at room temperature. The sample was then precipitated out using cold diethylether and Fmoc was deprotected using 30% piperidine in DMF. The sample was again precipitated out using cold diethylether. The precipitate was then solubilized in water and then dialyzed using a 2000Da MWCO Dialysis Casette to remove some by-products. **RP-HPLC:** PAMAM[8]-TXA was then purified using semi-preparative Thermo Hypersil GOLD C18 column (5µm, 250 x 10 mm) on 10 minutes 10 – 25% Sol B (methanol+0.1% TFA) gradient. The purified product was rotate evaporated, and the yield was 1% (0.4 mg). **MS QTOF (ESI+, 220V):** Mass found: 1272.3858 $[M+2H]^{2+}$, 848.5964 $[M+3H]^{3+}$, 636.7013 $[M+4H]^{4+}$; Calculated 1271.9173 $[M+2H]^{2+}$, 848.2806 $[M+3H]^{3+}$, 636.4550 $[M+4H]^{4+}$

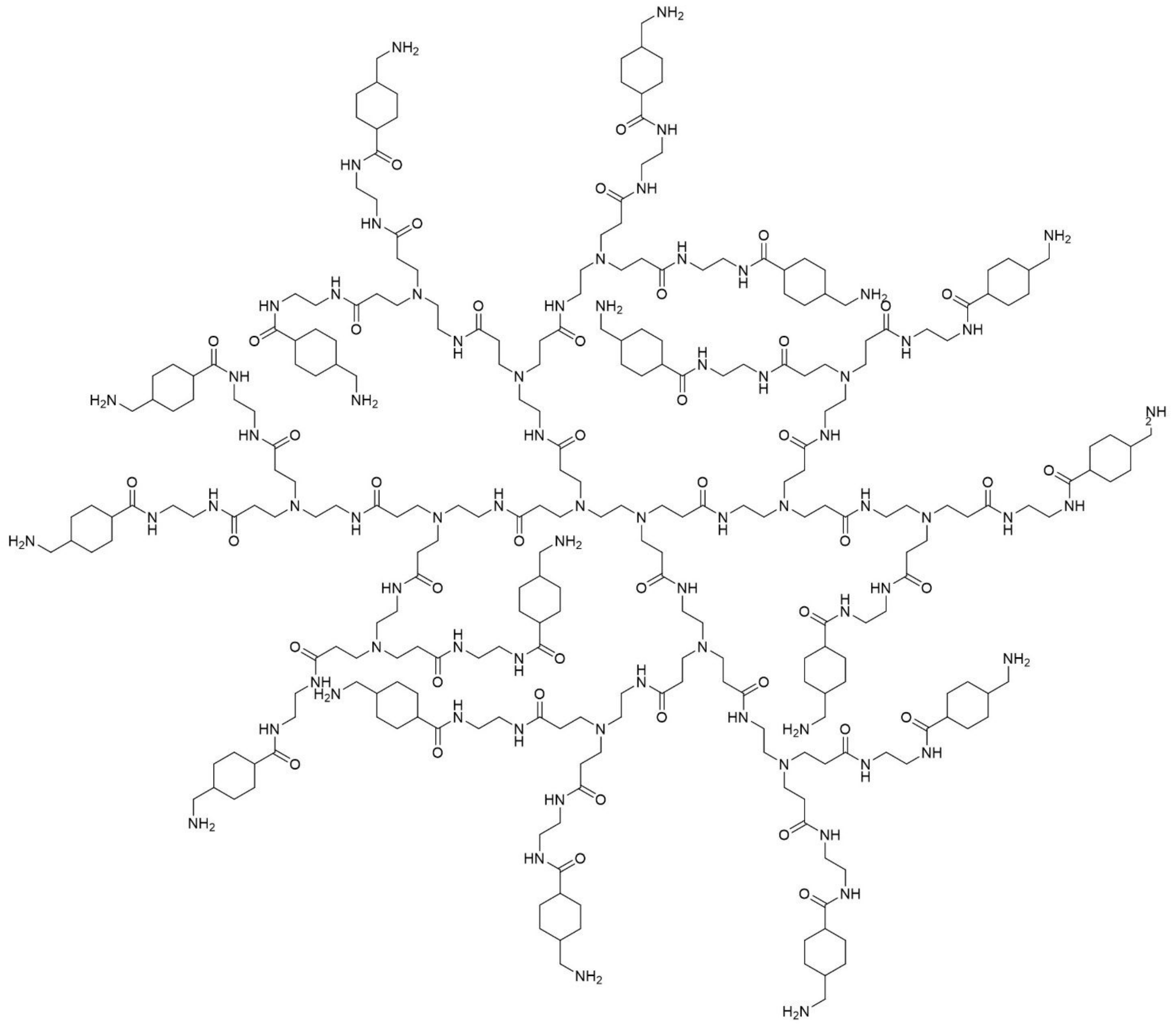

Chemical Formula: $C_{270}H_{496}N_{74}O_{44}$
Exact Mass: 5479.88
Molecular Weight: 5483.41

Figure S42: Chemical Structure and characterization of PAMAM[16]-TXA. PAMAM[16]-TXA was synthesized by reacting PAMAM[16] Dendrimer, Generation 2 (1 eq., 3 µmol) with Fmoc-TXA (20 eq., 61 µmol) using HBTU (18 eq., 55 µmol), Oxyma Pure (18 eq., 55 µmol) and DIEA (40 eq., 122 µmol) in DMF for 4 hours while stirring at room temperature. The sample was then precipitated out using cold diethylether and Fmoc was deprotected using 30% piperidine in DMF. The sample was again precipitated out using cold diethylether. The precipitate was then solubilized in water and then dialyzed using a 2000Da MWCO Dialysis Casette to remove some by-products. **RP-HPLC:** PAMAM[16]-TXA was then purified using semi-preparative Thermo Hypersil GOLD C18 column (5µm, 250 x 10 mm) on 10 minutes 20 – 25% Sol B (methanol+0.1% TFA) gradient. The purified product was rotate evaporated, and the yield was 3.4% (0.58 mg). **MS QTOF (ESI+, 220V):** Mass found: 1371.7356 $[M+4H]^{4+}$, 1097.5906 $[M+5H]^{5+}$,914.8270 $[M+6H]^{6+}$; Calculated 1370.9700 $[M+4H]^{4+}$, 1096.9760 $[M+5H]^{5+}$, 914.3133 $[M+6H]^{6+}$

S6 Inhibition Assays for Homo-multivalent TXA Inhibitors

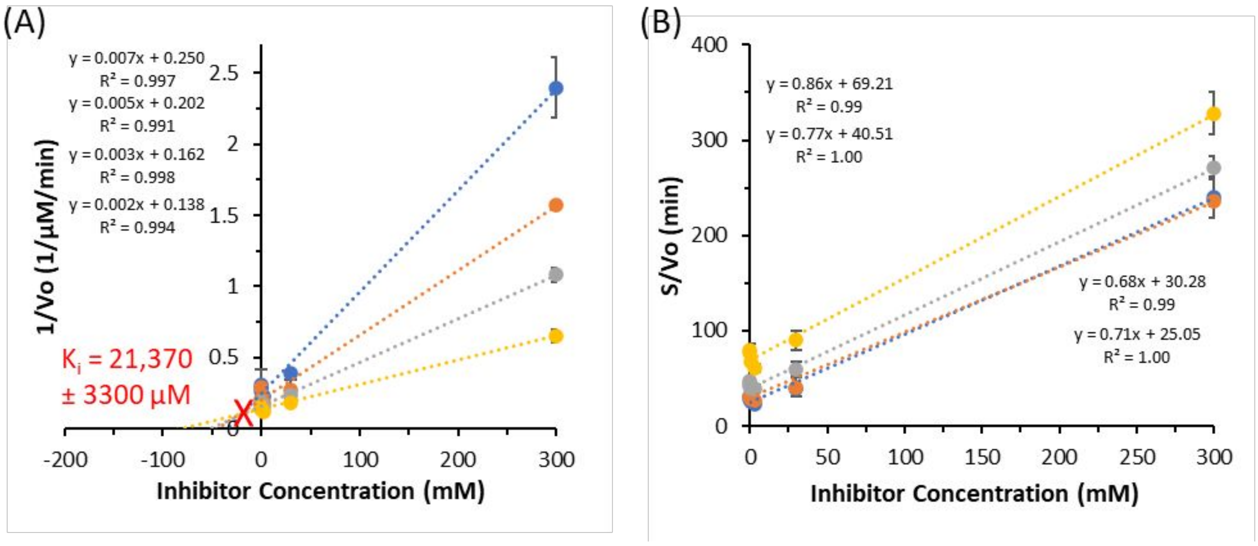

Figure S43: (A) K_i determination of TXA with plasmin utilizing a Dixon Plot. 0 – 300,000 µM of TXA was incubated with a fixed plasmin concentration of 42.5 nM in PBS pH 7.4. Four different S-2251 concentrations of 100 µM (blue), 150 µM (orange), 250 µM (gray), and 500 µM (yellow) were utilized to obtain K_i which is the negative intersection of the lines at 21,370 ± 3,300 µM. (B) Cornish-Bowden S/Vo vs I plot was used to determine the mode of inhibition. TXA was found to be a weak competitive inhibitor as the lines in this plot are parallel. All data is represented as mean ± SD of triplicate experiments.

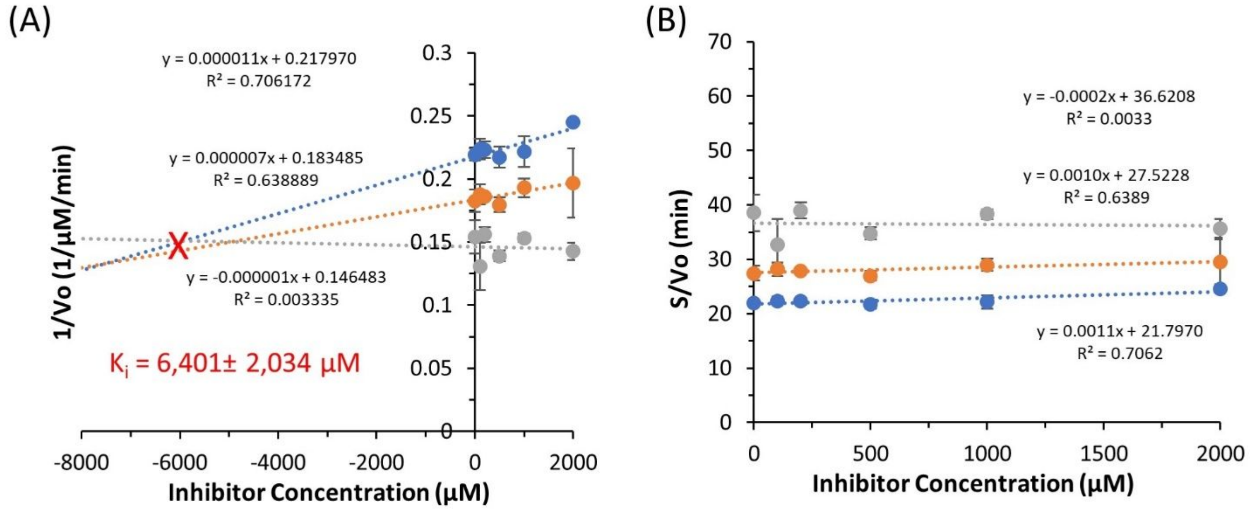

Figure S44: (A) K_i determination of Bis-TXA with plasmin utilizing a Dixon Plot. 0 – 2000 µM of Bis-TXA was incubated with a fixed plasmin concentration of 42.5 nM in PBS pH 7.4. Three different S-2251 concentrations of 100 µM (blue), 150 µM (orange) and 250 µM (gray) were utilized to obtain K_i which is the negative intersection of the lines at 6,401 ± 2,034 µM. (B) Cornish-Bowden S/Vo vs I plot was used to determine the mode of inhibition. Bis-TXA was found to be a weak competitive inhibitor as the lines in this plot are parallel. All data is represented as mean ± SD of triplicate experiments.

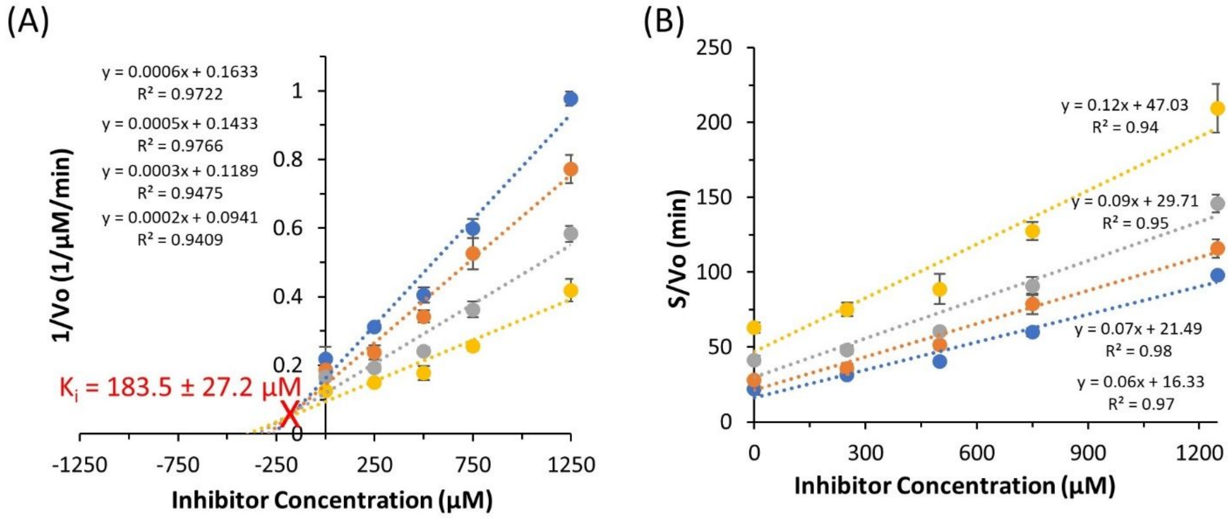

Figure S45: (A) K_i determination of PAMAM[4]-TXA with plasmin utilizing a Dixon Plot. 0 – 1250 µM of PAMAM[4]-TXA was incubated with a fixed plasmin concentration of 42.5 nM in PBS pH 7.4. Four different S-2251 concentrations of 100 µM (blue), 150 µM (orange), 250 µM (gray), and 500 µM (yellow) were utilized to obtain K_i which is the negative intersection of the lines at 183.5 ± 27.2 µM. (B) Cornish-Bowden S/Vo vs I plot was used to determine the mode of inhibition. PAMAM[4]-TXA was found to be a competitive inhibitor as the lines in this plot are parallel. All data is represented as mean ± SD of triplicate experiments.

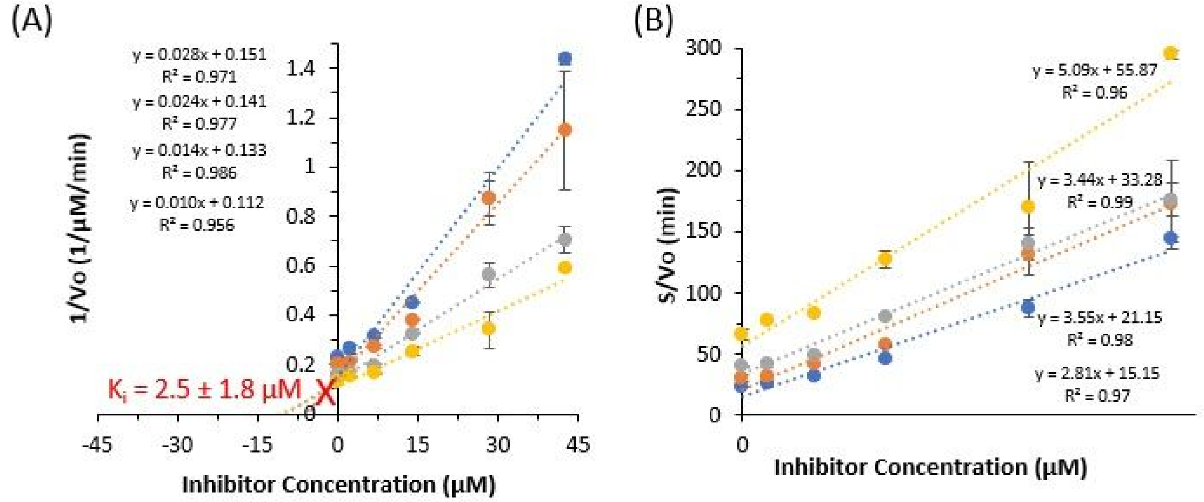

Figure S46: (A) K_i determination of PAMAM[8]-TXA with plasmin utilizing a Dixon Plot. 0 – 42 µM of PAMAM[8]-TXA was incubated with a fixed plasmin concentration of 42.5 nM in PBS pH 7.4. Four different S-2251 concentrations of 100 µM (blue), 150 µM (orange), 250 µM (gray), and 500 µM (yellow) were utilized to obtain K_i which is the negative intersection of the lines at 2.5 ± 1.8 µM. (B) Cornish-Bowden S/Vo vs I plot was used to determine the mode of inhibition. PAMAM[8]-TXA was found to be a competitive inhibitor as the lines in this plot are parallel. All data is represented as mean ± SD of triplicate experiments.

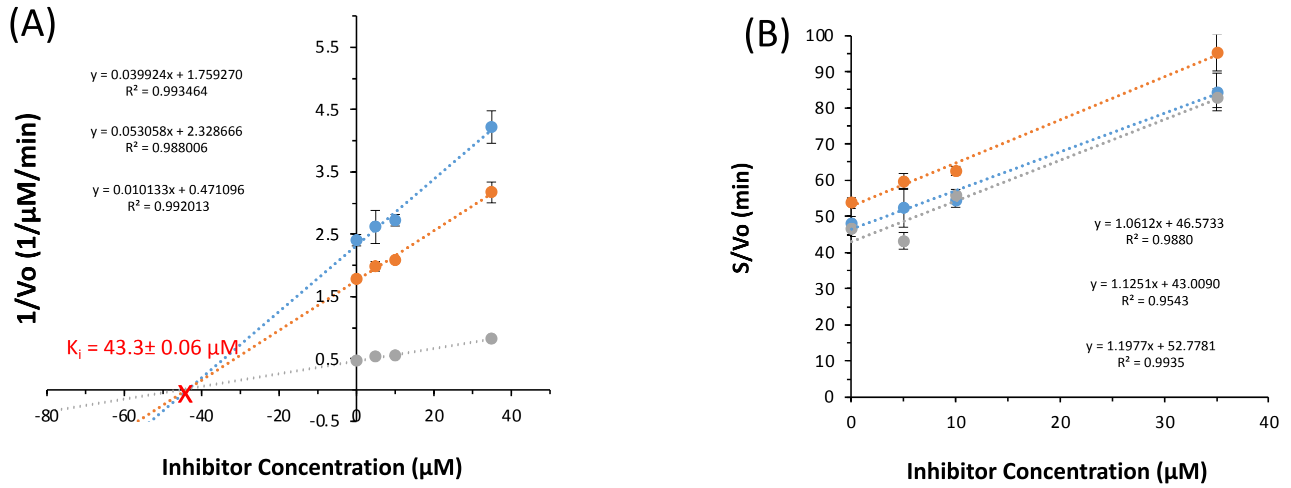

Figure S47: (A) K_i determination of PAMAM[8]-TXA with tPA utilizing a Dixon Plot. 0 – 35 µM of PAMAM[8]-TXA was incubated with a tPA concentration of 75 nM in PBS pH 7.4. Three different S-2288 concentrations of 20 µM (blue), 30 µM (orange) and 100 µM (gray), were utilized to obtain K_i which is the negative intersection of the lines at 43.3 ± 0.06 µM. (B) Cornish-Bowden S/Vo vs I plot was used to determine the mode of inhibition. PAMAM[8]-TXA was found to be a competitive inhibitor as the lines in this plot are parallel. All data is represented as mean ± SD of triplicate experiments.

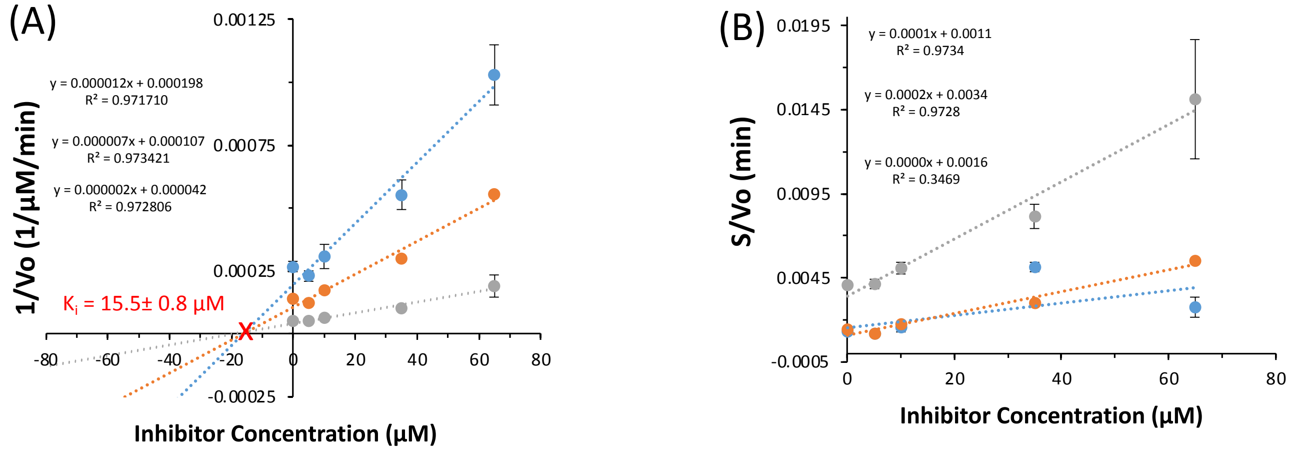

Figure S48: (A) K_i determination of PAMAM[8]-TXA with thrombin utilizing a Dixon Plot. 0 – 65 µM of PAMAM[8]-TXA was incubated with a fixed thrombin concentration of 0.25 U/mL in PBS pH 7.4 at 10% DMSO. Three different TSIII concentrations concentrations of 20 µM (blue), 30 µM (orange), and 100 µM (gray) were utilized to obtain K_i which is the negative intersection of the lines at 15.5 ± 0.06 µM. (B) Cornish-Bowden S/Vo vs I plot was used to determine the mode of inhibition. PAMAM[8]-TXA was not found to be a solely competitive inhibitor with potential allosteric contributions as the lines in this plot are not parallel. All data is represented as mean ± SD of triplicate experiments.

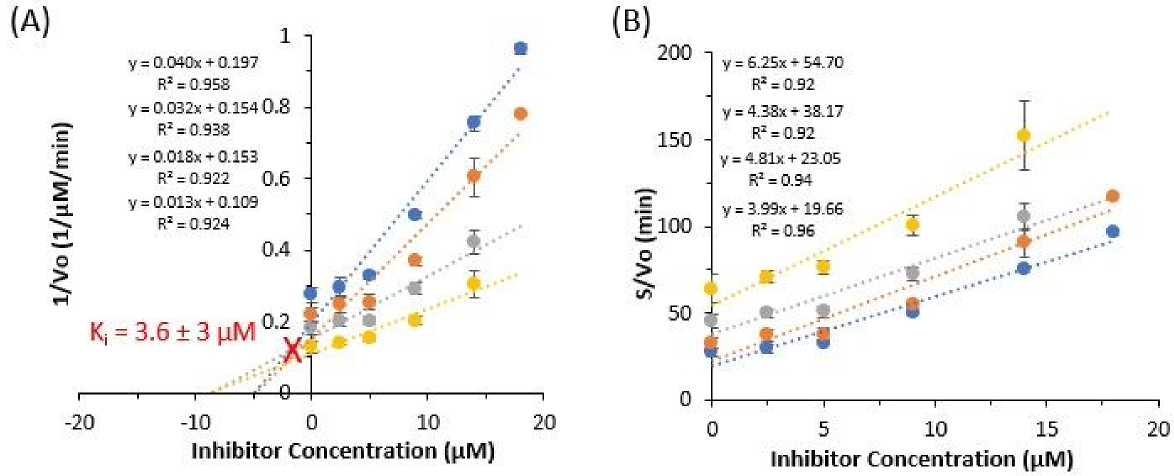

Figure S49: (A) K_i determination of PAMAM[16]-TXA with plasmin utilizing a Dixon Plot. 0 – 18 µM of PAMAM[16]-TXA was incubated with a fixed plasmin concentration of 42.5 nM in PBS pH 7.4. Four different S-2251 concentrations of 100 µM (blue), 150 µM (orange), 250 µM (gray), and 500 µM (yellow) were utilized to obtain K_i which is the negative intersection of the lines at 3.6 ± 3 µM. (B) Cornish-Bowden S/Vo vs I plot was used to determine the mode of inhibition. PAMAM[16]-TXA was found to be a competitive inhibitor as the lines in this plot are parallel. All data is represented as mean ± SD of triplicate experiments.

S7 Annular Fibrin Clot Assays

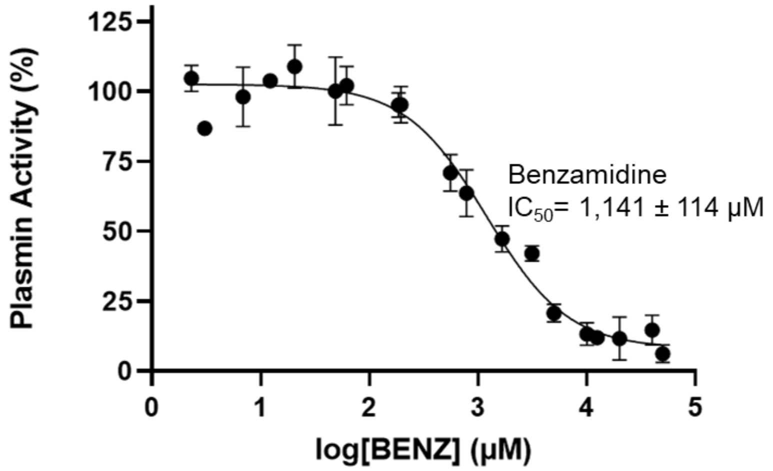

Figure S50: IC_{50} with benzamidine. Dose-response curve of plasmin (0.85 µM) with benzamidine (0 - 100,000 µM) yielded an IC_{50} of 1,141 ± 114 µM in an annular fibrin clot assay.

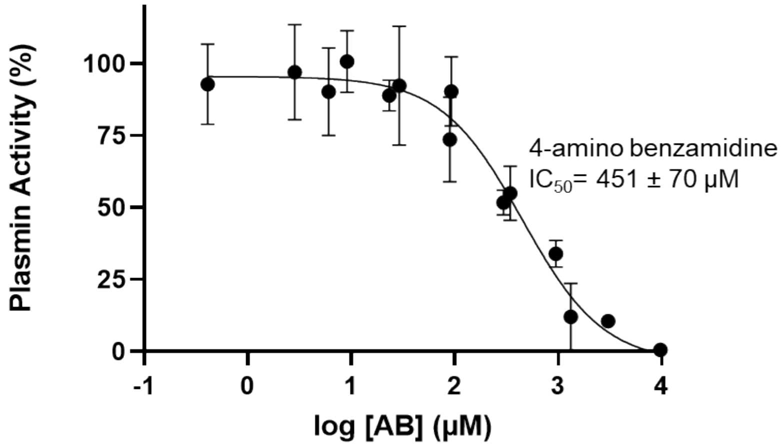

Figure S51: IC_{50} with 4-amino benzamidine. Dose-response curve of plasmin (0.85 µM) with 4-amino benzamidine (0 -10,000 µM) yielded an IC_{50} of 451 ± 70 µM in an annular fibrin clot assay.

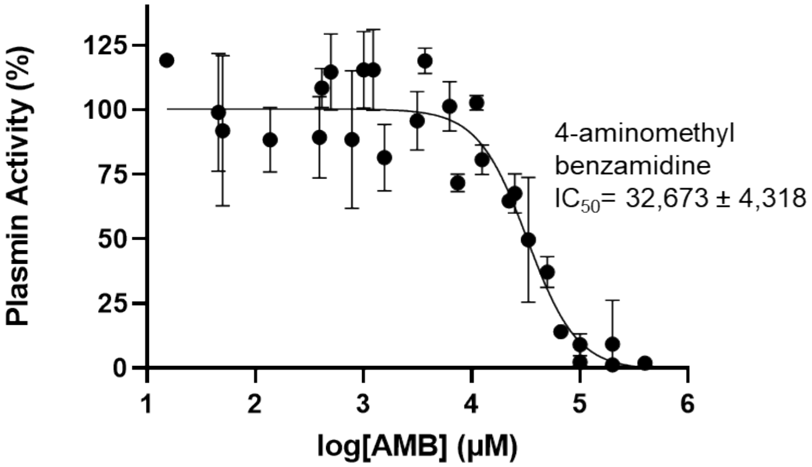

Figure S52: IC_{50} with 4-aminomethyl benzamidine. Dose-response curve of plasmin (0.85 µM) with 4-aminomethyl benzamidine (0 -1,000,000 µM) yielded an IC_{50} of 32,673 ± 4,318 µM in an annular fibrin clot assay.

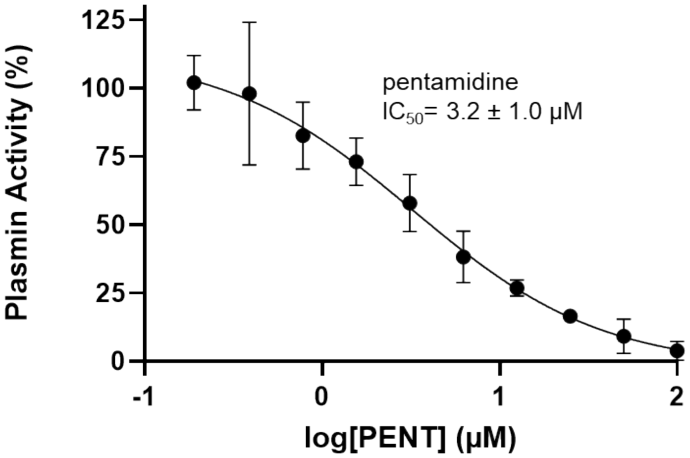

Figure S53: IC_{50} with pentamidine. Dose-response curve of plasmin (0.85 µM) with pentamidine (0 -100 µM) yielded an IC_{50} of 3.2 ± 1.0 µM in an annular fibrin clot assay.

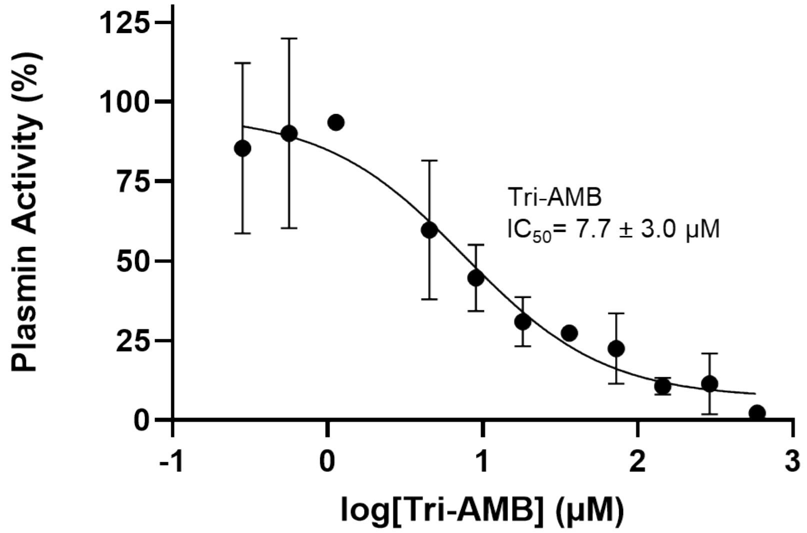

Figure S54: IC_{50} with Tri-AMB. Dose-response curve of plasmin (0.85 µM) with Tri-AMB (0 -1000 µM) yielded an IC_{50} of 7.7 ± 3.0 µM in an annular fibrin clot assay.

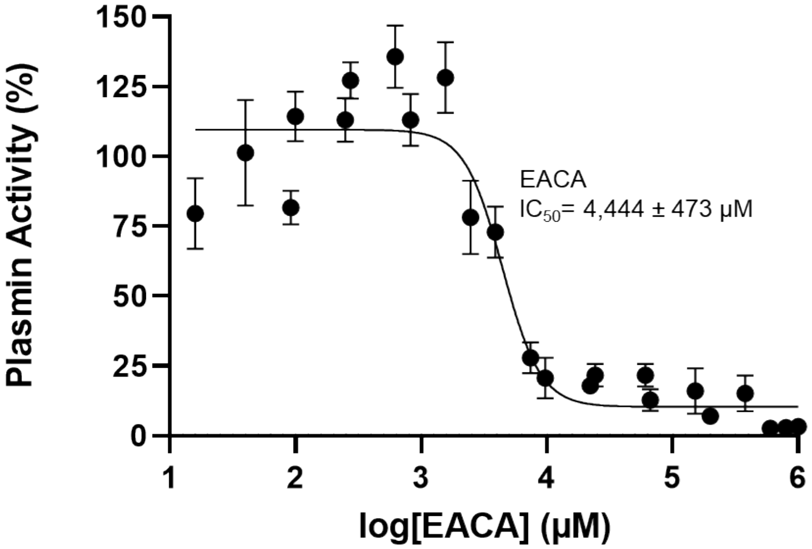

Figure S55: IC_{50} with EACA. Dose-response curve of plasmin (0.85 µM) with EACA (0 -1,000,000 µM) yielded an IC_{50} of 4,444 ± 473 µM in an annular fibrin clot assay.

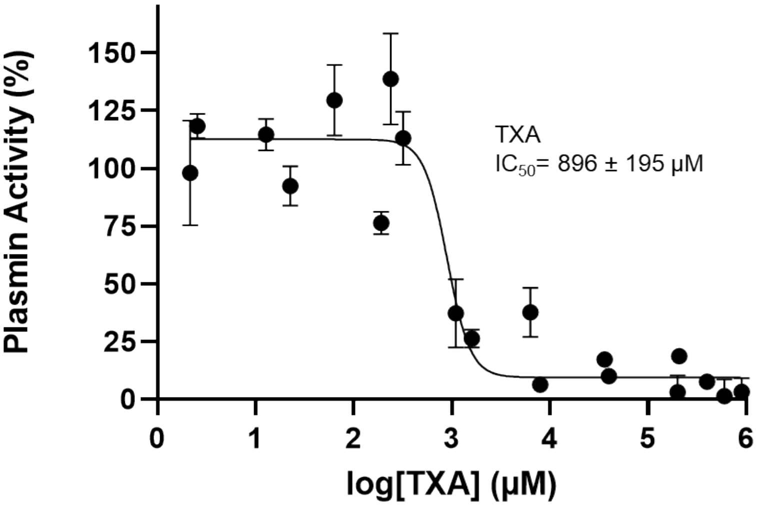

Figure S56: IC_{50} with TXA. Dose-response curve of plasmin (0.85 µM) with TXA (0 -1,000,000 µM) yielded an IC_{50} of 896 ± 195 µM in an annular fibrin clot assay.

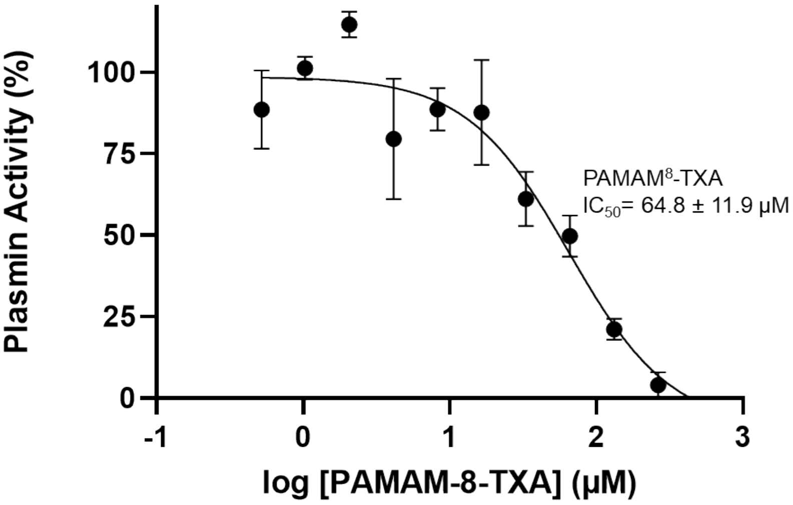

Figure S57: IC_{50} with PAMAM^{8}-TXA. Dose-response curve of plasmin (0.85 µM) with PAMAM^{8}-TXA (0 - 100 µM) yielded an IC_{50} of 64.8 ± 11.9 µM in an annular fibrin clot assay.

S8 Table

Table S1 Inhibition constants (K_i), relative potency rp^{AMB} (K_i^{AMB}/ K_i^{hetero}) and rp^{TXA} (K_i^{TXA}/ K_i^{hetero}) values of hetero-bivalent inhibitors with plasmin along with separation lengths.

**Inhibitor**	**K_i (μM)**	**rp^{AMB}**	**rp^{TXA}**	**Length (nm)**
AMB	1074 ± 19	-	-	1.0
TXA	21,370 ± 3,300	-	-	1.0
TXA-dPEG$_4$-AMB	42 ± 12	26	509	4.0
TXA-dPEG$_8$-AMB	99 ± 0.9	11	216	5.5
TXA-dPEG$_{12}$-AMB	207 ± 4.1	5	103	7.1
TXA-dPEG$_{36}$-AMB	75 ± 4.3	14	285	16.6
